# Dextran vs. Crystalloid Priming Solution in Cardiac Surgery: A Randomized Trial on Acute Kidney Injury

**DOI:** 10.1111/aas.70139

**Published:** 2025-10-27

**Authors:** Oskar Juvakka, Andreas Wallinder, Peter Hasse Møller‐Sørensen, Klaus Matschke, Anders Jeppsson, Lukas Lannemyr

**Affiliations:** ^1^ Department of Anesthesiology and Intensive Care Medicine, Institute of Clinical Sciences at the Sahlgrenska Academy University of Gothenburg Sweden; ^2^ Department of Cardiothoracic Anaesthesia Sahlgrenska University Hospital Gothenburg Sweden; ^3^ XVIVO Perfusion Gothenburg Sweden; ^4^ Department of Cardiothoracic Anesthesiology, Rigshospitalet Copenhagen University Hospital Copenhagen Denmark; ^5^ Klinik für Herzchirurgie, Herzzentrum Dresden GmbH Dresden Germany; ^6^ Department of Molecular and Clinical Medicine, Institute of Medicine, Sahlgrenska Academy University of Gothenburg Gothenburg Sweden; ^7^ Department of Cardiothoracic Surgery Sahlgrenska University Hospital Gothenburg Sweden

## Abstract

**Background:**

Acute kidney injury (AKI) is a frequent complication following cardiac surgery involving cardiopulmonary bypass (CPB). This is partly attributable to crystalloid‐based priming solutions causing both hemolysis and loss of oncotic pressure with tissue edema. While colloids like albumin and starches have not shown clear benefits, pilot studies using dextran‐based priming reported improved oncotic pressure, reduced hemolysis, and lower levels of a renal injury marker, suggesting potential renal protective effects.

**Objective:**

We hypothesized that a dextran‐based priming solution can reduce the incidence of postoperative AKI in high‐risk patients undergoing cardiac surgery with CPB.

**Methods:**

In this randomized, controlled, double‐blinded, multicenter trial, adult patients with a calculated postoperative AKI risk of ≥ 50% were assigned to receive either a dextran or a crystalloid‐based CPB priming solution. The primary outcome was the incidence of AKI within 96 h postoperatively. Secondary outcomes included perioperative hemolysis, net fluid balance, and the need for postoperative renal replacement therapy.

**Results:**

The trial was terminated early due to slow enrolment, with 101 of the planned 366 patients recruited. A total of 92 patients were included in the final analysis (43 in the dextran group, 49 in the control group). Postoperative AKI occurred in 81% and 53% of patients in the dextran and control groups, respectively (risk ratio 1.53, 95% confidence interval 1.15–2.06, *p* = 0.004). The dextran group demonstrated lower intraoperative hemolysis and a more favorable net fluid balance. Postoperative renal replacement therapy was required in 7% of the dextran group and 4% of the control group (*p* = 0.66). No significant differences in adverse events were observed between the groups.

**Conclusion:**

In high‐risk patients undergoing cardiac surgery with CPB, the use of a dextran‐based priming solution was associated with a significantly increased risk of postoperative AKI.

**Editorial Comment:**

This randomized multicenter trial compared dextran to a crystalloid‐based priming solution during cardiopulmonary bypass in participants with elevated risk of acute kidney injury. While the trial had to be terminated due to slow enrolment after about a third of planned cases were included, acute kidney injury was significantly more common in the dextran group, contrary to the primary hypothesis of the study. The study highlights the complexity and logistical challenges of conducting randomized treatment protocols for cardiopulmonary bypass, but at the same time highlights the importance of conducting such studies.

**Trial Registration:**

ClinicalTrials.gov identifier: NCT04293744

## 1 Introduction

Cardiac surgery associated acute kidney injury (AKI) affects up to one‐third of the patients and is associated with increased morbidity, mortality, and costs [1]. The use of cardiopulmonary bypass (CPB) contributes to AKI through several mechanisms such as inflammation, oxidative stress, microembolization, and hypoperfusion [2]. Another contributing factor is probably loss of oncotic pressure causing tissue edema and changes in blood rheology contributing to hemolysis, in part attributable to the use of crystalloid priming solutions [3, 4, 5]. In an effort to counteract these effects, colloid‐based priming solutions such as albumin and synthetic starches have been investigated, although none have demonstrated clear superiority over crystalloid solutions in terms of clinical outcomes [6, 7]. Current European guidelines recommend balanced crystalloid solutions as first‐line priming fluid, with selective addition of albumin, for example, to maintain oncotic pressure in high‐risk patients [8].

In a single center study, a priming solution based on the colloid dextran was compared to crystalloid priming solution. The dextran‐based priming improved colloid oncotic pressure and reduced hemolysis and the levels of a renal tubular injury marker (*N*‐acetyl‐β‐d‐glucosaminidase) [9, 10]. Based on these findings, which suggest a potential renal protective effect, we conducted this trial with the aim to compare the incidence of AKI within 96 h after cardiac surgery in patients receiving dextran‐based versus crystalloid‐based priming. We hypothesized that dextran‐based priming would reduce the incidence of postoperative AKI.

## 2 Methods

### 2.1 Study Design

The present study was a prospective international multicenter double‐blinded, randomized controlled trial comparing dextran‐based priming solution with standard crystalloid priming solution (in a 1:1 ratio) for CPB in patients with a high risk of AKI undergoing cardiac surgery. The study was approved by the Swedish Ethical Review Authority, dnr 2019‐06457, date of approval February 20, 2020, and was performed in compliance with the Declaration of Helsinki and consistent with ISO 14155:2020 and applicable regulatory requirements. The study was registered at ClinicalTrials.gov on January 17, 2020, with identifier NCT04293744. Written consent was obtained from all subjects before any study activity.

The participating centers were three tertiary cardiac surgery centers in Sweden, Denmark, and Germany. The study was initially started in Sweden in February 2020, but because of slow enrolment, the study was expanded to include patients in Denmark and Germany. An electronic Case Report Form was used for all data collection.

A detailed protocol (Clinical Investigation Plan) is included in Data S1.

All abbreviations used are presented in Table 1.

**TABLE 1:** Abbreviations.

AE	adverse events
AKI	acute kidney injury
CABG	coronary artery bypass graft
CPB	cardiopulmonary bypass
eGFR	estimated glomerular filtration rate
KDIGO	kidney disease improving global outcomes
NO	nitric oxide
PfHb	plasma‐free hemoglobin
SAE	serious adverse advent
SOC	standard of care
SvO_2_	mixed venous oxygen saturation

### 2.2 Patients

Patients aged ≥ 18 years, scheduled for elective or urgent cardiac surgery requiring the use of CPB, were prospectively screened for eligibility. Only patients with a predicted risk of ≥ 50% of developing postoperative AKI were included. To assess the risk of AKI, the “Acute Kidney Injury Risk Score” was used. This is a predictive model that can be used in clinical trials to enable enrolment of patients with a high event rate, thereby increasing study power [11]. The cut‐off value used, correlating with a 50% risk of AKI, was based on a previous study [12]. Exclusion criteria were emergency surgery, inability to give informed consent, known intolerance or contraindication to dextran, ongoing sepsis or endocarditis, and preoperative dialysis. In addition, patients with known bleeding disorders and patients in whom antithrombotic medications were not discontinued per institutional protocol were excluded.

### 2.3 Randomization and Blinding

An online module was used to randomize patients to dextran or standard of care (SOC) in a 1:1 ratio, with allocation stratified for eGFR < 60 mL/min and an expected CPB time > 60 min. All personnel assigned to the case were blinded. A perfusionist, not assigned to the case, performed the randomization the same day as the planned surgery. The same perfusionist did the priming of the CPB circuit and then had no further involvement in the case. A new perfusionist, blinded to the priming solution, was thereafter responsible for the management of CPB perioperatively. Furthermore, the patient and the surgical and anesthesia teams responsible for the patient throughout the surgery and perioperative care were blinded to the intervention.

### 2.4 Intervention and CPB Management

The priming solution (PrimECC, XVIVO Perfusion, Gothenburg, Sweden) used in the dextran group is a CE‐marked physiological salt solution containing dextran 1 and dextran 40. The SOC group received priming with Ringer's acetate and mannitol. The priming volume was same in both groups and was set to the volume used in standard care in respective participating center, that is, a volume between 1000 and 1500 mL. Retrograde autologous priming was not allowed in the study. After heparinization, non‐pulsatile CPB was performed at a flow of 2.4–2.6 L/min/m^2^, with a target mixed venous oxygen saturation (SvO_2_) > 70%. In patients with a hematocrit < 24% and SvO_2_ < 70%, red blood cell transfusion or hemofiltration was allowed. After weaning from CPB, the remaining CPB volume was transfused to the patient either directly through the arterial cannula, through an autotransfusion device or through a regular bag containing whole blood.

### 2.5 Outcomes

The primary outcome was incidence of any grade of AKI defined according to the KDIGO criteria [13]; an increase of S‐Cr ≥ 27 μmol/L within 48 h or ≥ 50% from baseline within 96 h after surgery.

Secondary outcomes were postoperative development of S‐Cr, eGFR (according to CKD‐EPI formula), AKI grade (according to KDIGO criteria), use of renal replacement therapy (RRT), hemolysis (measured as plasma‐free hemoglobin [PfHb]) and perioperative net fluid balance. Measurements were made after induction of anesthesia, after 60 min of CPB, 60 min after termination of CPB, and at 24 ± 3, 48 ± 3, 72 ± 3, and 96 ± 3 h after surgery. In addition, liver enzymes aspartate aminotransferase and alanine aminotransferase, and markers of coagulation including international normalized ratio (INR), activated partial thromboplastic time (APTT), thrombocyte count, and fibrinogen levels were measured 24 h after surgery.

Intraoperative and postoperative parameters including type of surgery, operative priority, CPB time, aortic cross‐clamp time, use of vasopressors, fluid balance including infusions of crystalloids and colloids, transfusions, bleeding and urine output were registered. Analyses of cerebral injury markers and urine renal tubular injury markers were planned but not executed due to the early termination of the study.

Adverse events (AEs) were registered and defined as any adverse medical change, that is, de novo or increased severity in a preexisting condition, from the subject's baseline condition that occurred during the clinical study, whether considered related to the intervention or not. Serious adverse events (SAEs) were registered in the same manner and were defined as either death or a serious deterioration in the health condition of the subject. AKI was considered an outcome variable and not an AE.

### 2.6 Sample Size Calculation

The power analysis was based on a previous study on the dextran solution [10] and a study population with a predicted 50% risk of postoperative AKI. For the sample size calculation, we hypothesized that the intervention would reduce the incidence of postoperative AKI by 30% (i.e., from 50% to 35%). With a power of 80% and an *α* = 0.05, a total of 366 patients was needed to demonstrate this treatment effect of 30%.

### 2.7 Statistical Analysis

The main analysis was performed on the intention to treat population and as randomized. A supplemental as treated analysis was made on the per‐protocol population. Descriptively, continuous variables were presented by mean with standard deviation or median with range and categorical variables by counts and percentages.

For test between two groups, Fisher's exact test was used for dichotomous variables, Mantel–Haenszel chi‐square trend test for the ordered categorical variables, chi‐square test for non‐ordered categorical variables, and Mann–Whitney *U* test or two‐sample *t*‐test for continuous variables depending on the variable distribution. For the primary variable, effect size was described by risk ratio with 95% CI. A *p* value of 0.05 was considered significant for the confirmatory primary analysis. AEs were analyzed according to the safety population, that is, as treated.

## 3 Results

### 3.1 Patients

**FIGURE 1:**
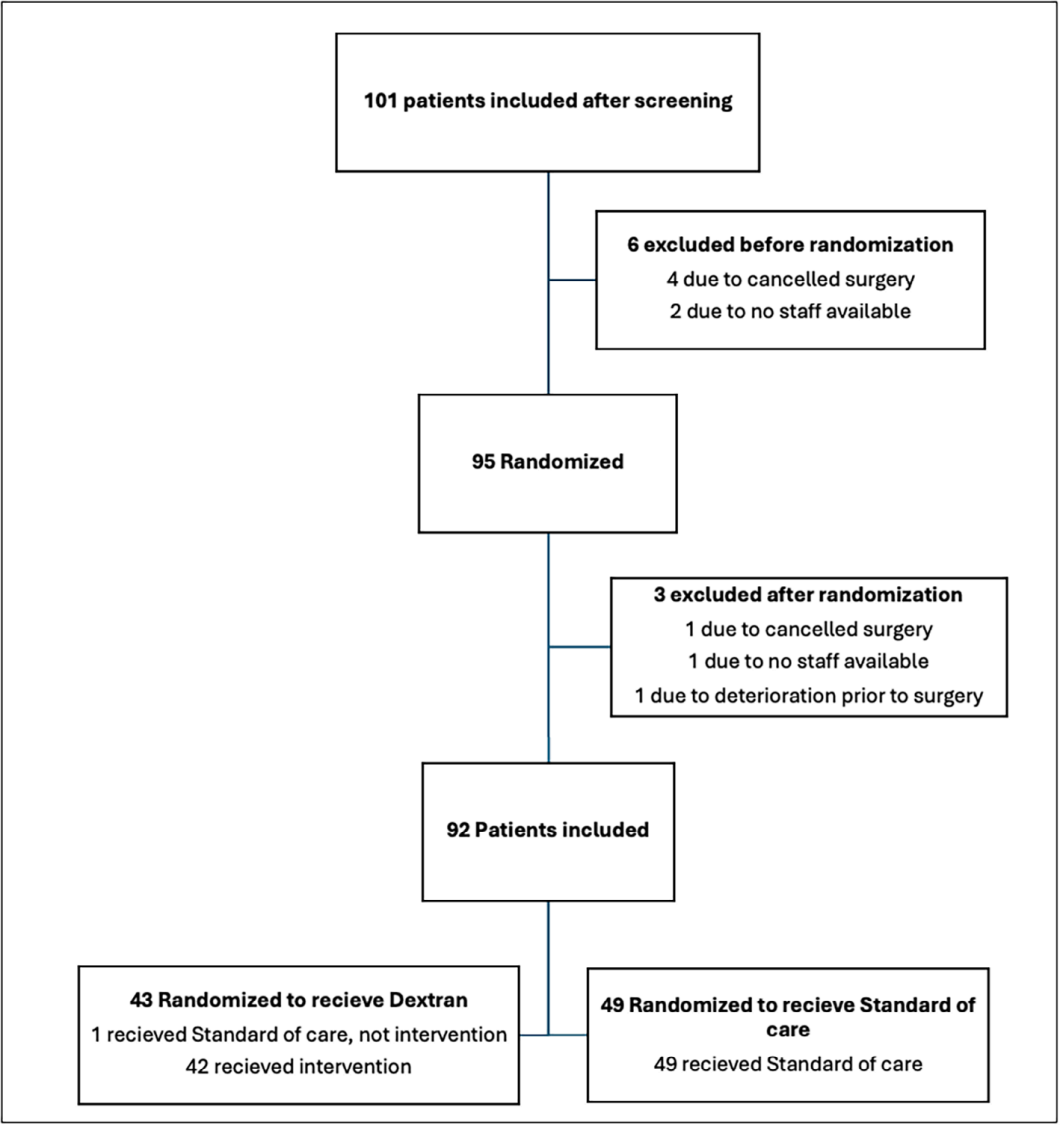
Consort diagram.

Three university hospital cardiac surgery centers participated in the study. From February 2020, patients who underwent cardiac surgery were screened for eligibility. The inclusion/screening ratio was 1/15. The study was temporarily paused at different intervals due to the covid pandemic. Forty months after the inclusion of the first patient, the study was stopped by the sponsor for further inclusions due to a slow recruitment rate. At this time, a total of 101 patients had been enrolled after screening (Figure 1). Six patients were excluded before randomization (4 due to canceled surgery; 2 due to lack of available staff for blinded randomization). Three patients were excluded after randomization but before any intervention (1 due to canceled surgery; 1 due to no staff available for priming CPB; 1 patient due to deterioration before surgery).

In total, 92 patients were included in the analysis, 43 (46.7%) in the dextran group; 49 (53.3%) in the SOC group. Forty‐two of forty‐three patients in the dextran group received the dextran‐based priming solution (one received SOC); all patients in the control group received SOC. The consort diagram is depicted in Figure 1.

Baseline characteristics including demographic variables, kidney function, AKI risk score, and medical history were comparable between the groups (Table 2).

**TABLE 2:** Baseline characteristics.

	Dextran (*n* = 43)	SOC (*n* = 49)
Male gender	41 (95.3)	42 (85.7)
Age, years	73.3 ± 5.7	72.3 ± 6.3
BMI, kg/m^2^	29.2 ± 5.2	29.5 ± 5.2
Current smoker	5 (11.6)	6 (12.6)
Diabetes mellitus	22 (51.2)	21 (42.9)
Peripheral vascular disease	7 (16.3)	9 (18.4)
Hypertension	41 (95.3)	48 (98.0)
Left ventricular ejection fraction, %	50.1 ± 11.1	48.3 ± 13.9
S‐creatinine, μmol/L	107.8 ± 33.8	105.1 ± 24.5
eGFR, mL/min/1.73m^2^	63.0 ± 22.1	61.7 ± 18.5
Preoperative acute kidney injury risk score	38.4 ± 9.4	40.1 ± 11.1

*Note:* Data are presented as mean ± standard deviation or number of observations (percentage). Statistical comparisons: Fisher's exact test for categorical variables and Mann–Whitney *U* test for continuous variables.

Abbreviations: BMI, body mass index; eGFR, estimated glomerular filtration rate; SOC, standard of care.

### 3.2 Intraoperative Data

Coronary artery bypass grafting (CABG) with or without valve procedures was performed in 34 of 43 (79%) and 25 of 49 (51%) in the dextran and SOC groups, respectively. Isolated valve procedures were done in 7 of 43 (16%) versus 17 of 49 (35%), and other open cardiac surgery procedures requiring CPB were done in 2 of 43 (5%) versus 7 of 49 (14%) in the dextran and SOC groups, respectively. In the dextran group, 20 of 43 (47%) were urgent operative priority cases versus 14 of 49 (29%) in the SOC group. The type of surgery is presented in Table S1.

The median duration of CPB was 1.8 [0.8–7.3] versus 1.4 [0.9–8.4] hours, and cross‐clamp time was 1.3 [0.6–4.5] versus 1.1 [0.5–4.2] hours in the dextran and SOC groups, respectively.

The median intraoperative net fluid balance was almost 600 mL lower in the dextran group compared to the SOC group (*p* = 0.006) (Table 3). The volume of hemofiltration during CPB was skewed, with a median of 0 in both groups but a mean volume of 494 ± 990 versus 129 ± 473 in the dextran and SOC groups, respectively (*p* = 0.010). There were no differences between groups in the volume of priming solution, intraoperative bleeding, transfusions, intraoperative blood salvage use (Cell Saver), or urine production (Table 3).

**TABLE 3:** Intraoperative fluids and medication.

	Dextran (*n* = 43)	SOC (*n* = 48)	*p*
Priming solution during surgery, mL	1300 [1200–1600]	1300 [800–2500]	0.32
Net fluid balance during surgery, mL	1270 [−1710 to 6410]	1850 [−1920 to 6310]	0.006
Bleeding during surgery, mL	500 [100–12,800]	325 [100–3070]	0.30
Crystalloids during surgery, mL	1223 [0–4500]	1527 [0–4528]	0.33
Colloids during surgery, mL	0 [0–200]	0 [0–4040]	0.48
Erythrocyte transfusion during surgery, mL	0 [0–1750]	0 [0–750]	0.13
Plasma transfusion during surgery, mL	0 [0–2500]	0 [0–1200]	0.11
Thrombocyte transfusion during surgery, mL	0 [0–1600]	0 [0–1100]	0.08
Urine production during surgery, mL	390 [30–1900]	425 [30–1207]	0.30
Hemofiltration during CPB, mL	0 [0–4840]	0 [0–2600]	< 0.001
Inotropes during CPB	5 (11.6)	5 (10.2)	> 0.99
Vasopressors during CPB	18 (41.9)	16 (32.7)	0.39
Diuretics during CPB	1 (2.3)	2 (4.2)	> 0.99

*Note:* Data are presented as median with range or number of observations (percentage). Statistical comparisons: Fisher's exact test for categorical variables and Mann–Whitney *U* test for continuous variables.

Abbreviations: CPB, cardiopulmonary bypass; SOC, standard of care.

### 3.3 Outcome

**FIGURE 2:**
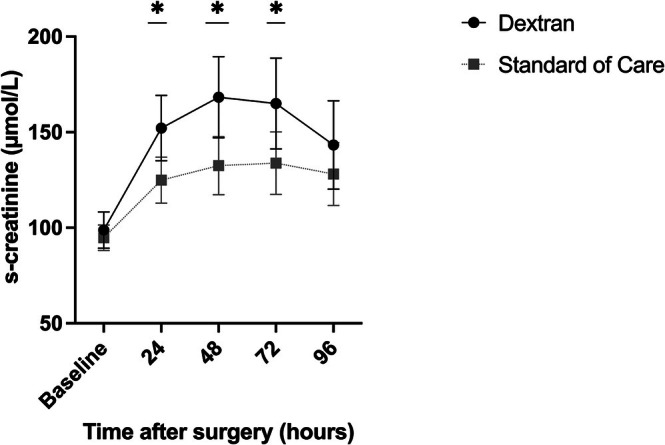
Serum creatinine development. Values are mean with 95% CI. *p* < 0.05. Statistical comparisons: Mann–Whitney *U* test.

The incidence of postoperative AKI within 96 h (primary outcome) was 81% (35/43 patients) in the dextran group compared to 53% (26/49 patients) in the SOC group (*p* = 0.004; risk ratio 1.53, 95% CI: 1.15–2.06). The serum‐creatinine levels did not differ between the groups before surgery but were significantly higher in the dextran group at all postoperative time points within 72 h after surgery (Figure 2), with corresponding lower eGFR. Nadir eGFR occurred at 48 h after surgery, with 43 ± 23 and 53 ± 22 mL/min/1.73 m^2^ in the dextran and SOC groups, respectively (*p* = 0.03).

**FIGURE 3:**
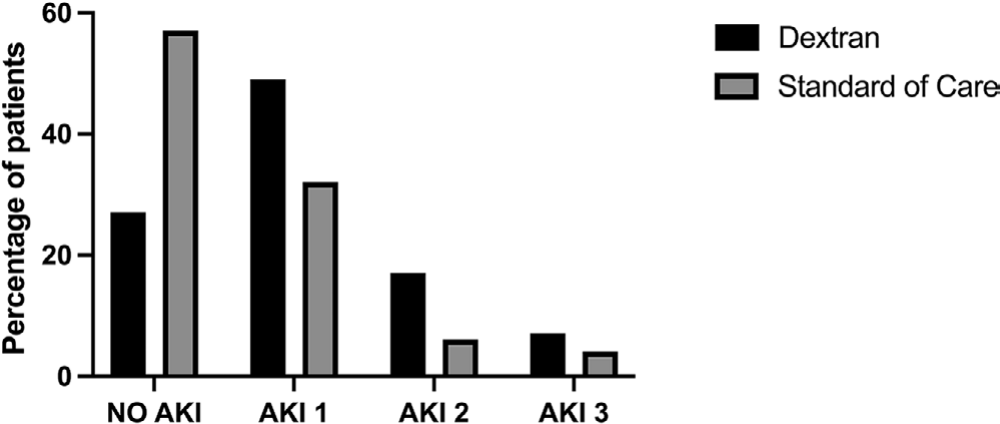
AKI stage at 48 h after surgery. Numbers are percentage of patients in the dextran (*n* = 41) and standard of care group (*n* = 47). Statistical comparisons: Mantel–Haenszel chi‐square trend test, *p* = 0.01 for group difference.

Figure 3 shows the distribution of AKI stages at 48 h after surgery, with significantly more cases of AKI Grades 1 and 2 in the dextran group, but equal numbers of AKI Grade 3. RRT within 96 h after surgery was required in 3 of 43 (7.1%) patients in the dextran group and 2 of 49 (4.2%) patients in the SOC group (*p* = 0.66). At hospital discharge, one patient in the dextran group had RRT versus no patient in the SOC group (*p* = 0.46).

Regarding hemolysis, the PfHb was lower in the dextran group at 60 min on CPB (0.18 ± 0.09 vs. 0.34 ± 0.23 g/L; *p* < 0.001). There were no significant differences in postoperative liver enzymes, INR, APTT, fibrinogen levels, or thrombocyte count between the groups.

There were no differences in the use of vasopressors, transfusion of blood products, or use of procoagulants within the first 24 h after surgery (Table S2).

### 3.4 Per‐Protocol Analysis

This population excluded patients with major protocol violations in each group; in total, 35 patients in the dextran group and 41 in the SOC group were included in this analysis. A summary of the per‐protocol population, major protocol violations, and all analyzed variables is available in Data S3.

The main findings were similar in this population as in the intention to treat population presented above. This included the incidence of AKI (86% vs. 54%, *p* = 0.003), net median intraoperative fluid balance (1275 mL [−1711 to 6406] vs. 2292 [−1917 to 6331], *p* = 0.006), and intraoperative hemolysis (PfHb 0.19 ± 0.09 g/L vs. 0.34 ± 0.23, *p* = 0.003) for the dextran and SOC group, respectively.

### 3.5 AEs

Adverse advents were analyzed as treated (dextran group *n* = 42, SOC group *n* = 50). At least one SAE occurred in 10 of 42 patients (23.8%) in the dextran group and 14 of 50 (28.0%) in the SOC group. The most common SAEs were postoperative respiratory failure in the dextran group (*n* = 2, 4.8%) and postoperative hemorrhage in the SOC group (*n* = 3, 6%). No anaphylactic reactions were reported in either study group.

SAE's resulting in death were observed in two patients in the dextran group; one due to postoperative biventricular cardiac failure and one due to postoperative dislocation of a mitral valve prosthesis, and one patient in the SOC group due to an intraoperative aortic dissection (*p* = 0.59). None of these deaths were assessed as related to the study intervention.

A detailed statistical report is included in Data S2.

## 4 Discussion

### 4.1 Main Finding

This randomized controlled trial tested the hypothesis that a dextran‐based priming solution for CPB would reduce the incidence of postoperative AKI in high‐risk cardiac surgery patients. Contrary to our hypothesis, we observed a significantly higher incidence of AKI in the dextran group compared to the crystalloid group.

### 4.2 Interpretation of Results

CPB is known to induce hemolysis, releasing PfHb, which scavenges nitric oxide (NO). Reduced NO availability impairs microcirculation and may contribute to renal hypoxia—a key mechanism in cardiac surgery associated AKI [4, 14]. Dextran increases blood viscosity and thus can decrease turbulent flow and shear stress, which may reduce hemolysis [5], and indeed, the dextran group had lower PfHb levels. Despite this, they paradoxically experienced more AKI, underscoring the multifactorial nature of renal injury in cardiac surgery.

The previous studies on dextran‐based priming showed reduced hemolysis and a lower level of a renal tubular injury marker [9, 10]. However, these studies involved patients at normal risk for AKI, with AKI incidences of 18% and 22% in the dextran and SOC groups, respectively. In contrast, our study targeted an enriched cohort with ≥ 50% predicted AKI risk using the AKI risk score, potentially revealing adverse effects of dextran not evident in lower‐risk populations.

Dextran‐induced AKI may result from several mechanisms, including tubular precipitation of dextran material [15, 16] and functional reductions in glomerular filtration due to increased colloid osmotic pressure [17].

Besides the impact of dextran, there are additional factors that may have influenced the study results. Despite randomization, there were near significant group differences in the types of surgery. A higher proportion of patients in the dextran group underwent CABG procedures. Coronary disease may be associated with renal artery stenosis [18], which could affect renal circulation negatively during CPB. However, evidence suggests that valve surgery is associated with a higher incidence of AKI compared to CABG, implying that the type of surgery is not the underlying cause of increased AKI in the dextran group [19]. Another important aspect is that the patients in the dextran group had numerically longer CPB duration, a known risk factor for AKI [1].

Interestingly, the SOC group had a more positive net fluid balance, consistent with prior findings [9]. While a higher perioperative hemodilution may lower serum creatinine, a positive fluid balance is associated with an increased risk of AKI during cardiovascular surgery [20]. Moreover, the dextran group had greater hemofiltration during CPB, possibly due to higher intravascular volume retention from the hyperoncotic solution. Although large‐volume hemofiltration has been linked to impaired renal outcomes, evidence remains inconclusive [21, 22].

When the study was commenced, mannitol was still added to the priming solution as a clinical routine, and therefore this priming composition was used throughout the study in the SOC group. Current CPB guidelines no longer recommend the use of mannitol in priming solutions [8] due to insufficient evidence supporting a beneficial effect on AKI [23]. In light of this, the inclusion of mannitol in the SOC group's priming solution is not expected to have had a major influence on AKI outcomes.

### 4.3 Strengths and Limitations

This was a multicenter, randomized, double‐blind trial with rigorous data collection and external monitoring, enhancing the reliability and generalizability of findings. However, the study was terminated early after the enrolment of 101 patients—due to the COVID‐19 pandemic and slow recruitment. Recruitment challenges were primarily related to the strict inclusion criterion requiring an AKI risk score corresponding to a ≥ 50% predicted risk of AKI; only 1 in 15 screened patients met this threshold. As a result, the final sample size was limited. Since the study was stopped before the planned enrolment had been reached, there was a loss of study power, emphasizing the need for precaution in the interpretation of our results.

The primary outcome was based on serum creatinine using KDIGO criteria, which has limited sensitivity and specificity. Urine output was not included, and creatinine was only monitored for 4 days postoperatively, potentially missing late‐onset AKI.

## 5 Conclusion

In high‐risk cardiac surgery patients, a dextran‐based CPB priming solution was associated with an increased incidence of AKI. These findings do not support the use of dextran‐based priming to improve renal outcomes and suggest potential harm in this vulnerable population.

## Author Contributions

**Oskar Juvakka:** writing – review and editing, writing – original draft, investigation, conceptualization, project administration, data curation, visualization. **Andreas Wallinder:** writing – review and editing, methodology, resources, conceptualization, supervision, funding acquisition. **Peter Hasse Møller‐Sørensen:** writing – review and editing, investigation, resources, project administration. **Klaus Matschke:** writing – review and editing, investigation, resources, project administration. **Anders Jeppsson:** writing – review and editing, conceptualization, methodology, supervision, project administration. **Lukas Lannemyr:** writing – review and editing, writing – original draft, supervision, methodology, investigation, formal analysis, data curation, conceptualization, project administration, visualization.

## Ethics Statement

The study was approved by the Swedish Ethical Review Authority, dnr 2019‐06457, date of approval February 20, 2020.

## Consent

Written consent was obtained from all subjects before any study activity.

## Conflicts of Interest

Anders Jeppsson reports personal fees for advisory boards and/or lectures from AstraZeneca, LFB Biotechnologies, Bayer, Boehringer‐Ingelheim, Novo Nordisk, and Werfen, outside the present work. Lukas Lannemyr reports consultancy fees from XVIVO Perfusion. Andreas Wallinder is an employee of XVIVO Perfusion. The other authors declare no conflicts of interest.

## Supporting information


**Data S1:** Supporting Information.

**Data S2:** Supporting Information.

**Data S3:** Supporting Information.

## Data Availability

The data that support the findings of this study are available from the corresponding author upon reasonable request.
