# Supplementary material for: Dextran vs. Crystalloid Priming Solution in Cardiac Surgery: A Randomized Trial on Acute Kidney Injury
Source: Acta Anaesthesiol Scand. 2025 Oct 27;70(1):e70139. doi: 10.1111/aas.70139 (PMC12558647; doi:10.1111/aas.70139)
Supplement: Supplementary file 1 — Data S1: Supporting Information. [file AAS-70-0-s003.pdf]

Table of Contents

1 Definitions and Abbreviations 10
2 Background 12
3 Pre-clinical data 13
4 Clinical data 13
5 Products to be evaluated 13
5.1 Test group 13
5.2 Control group 13
5.3 Volume of priming solutions 14
5.4 Method of priming 14
5.5 Instruction for use PrimECC® 14
5.6 Product distribution 17
5.7 Product accountability 18
6 Study Hypotheses and Objectives 18
6.1 Hypothesis 18
6.2 Primary objective 18
6.3 Secondary objective 18
6.4 Exploratory objective 18
6.5 Primary endpoint 18
6.6 Secondary endpoint 18
6.7 KDIGO creatinine criteria, staging of AKI 19
6.8 Exploratory endpoint 19
6.9 Other pre-specified outcome measures 19
6.10 Estimated sample size and power 19
7 Study Design 19
7.1 Risks and Benefits 20
7.1.1 Anticipated clinical benefits 20
7.1.2 Anticipated adverse device effects 20
7.1.3 Risks associated with participation in the clinical investigation 20
7.1.4 Risk/benefit assessment 20
7.2 Study Groups 20
7.2.1 Blinding procedure 20
7.2.2 Emergency unblinding 21

Etikprövningsmyndigheten
2023-00034-02-350928
2023-01-03

7.3 Study Subjects ... 21
7.4 Schedule of assessment ... 22
7.5 Timeline ... 23
8 Schedule of Assessments ... 23
8.1 Informed Consent ... 23
8.2 Prior to surgery ... 23
8.3 After induction of anesthesia ... 23
8.4 1- hour after induction of ECC ... 23
8.5 1-hour post-ECC ... 23
8.7 Postoperative day 2 (within 48 h after ECC) ... 24
8.8 Postoperative day 3 (within 72 h after ECC) ... 24
8.9 Postoperative day 4 (within 96 h after ECC) ... 24
8.10 Intraoperative parameters ... 24
8.11 Postoperative parameters ... 24
8.12 Discharge ... 24
8.13 Suspension or termination of the clinical investigation ... 24
9 Adverse events ... 24
9.1 Adverse event definition and reporting ... 24
9.2 AE determination ... 25
9.3 Serious adverse event ... 26
9.4 Adverse device effect ... 26
9.5 Serious adverse device effect ... 26
9.6 Unanticipated serious adverse device effect (USADE) ... 26
9.7 Reporting of all adverse events ... 27
9.8 Relationship of the AE to the study device or study procedure ... 27
9.9 Degree of severity ... 29
9.10 Significant infection ... 29
9.11 Device deficiency ... 29
9.12 Expedited reporting of serious adverse events and device deficiencies ... 29
10 Data collection and management ... 30
10.1 Peri- and postoperative renal function ... 31
10.2 Peri- And postoperative liver function ... 31
10.3 Peri- and postoperative pulmonary function ... 31
10.4 Peri and post-operative heart injury ... 31
10.5 CNS Injury ... 31

Etikprövningsmyndigheten
2023-00034-02-350928
2023-01-03

10.6 Peri- and postoperative Hemolysis 31
10.7 ICU Length of stay 31
10.8 Hospital length of stay 31
11 Statistical analysis plan 31
11.1 Populations for analysis 32
11.2 Patient demographics/ other baseline characteristics 32
11.3 Analysis of the primary variable 32
11.4 Analysis of secondary objective 32
11.5 Analysis of exploratory variable 33
11.6 Safety 33
11.7 Missing Data 33
11.8 Protocol Deviations 33
12 Monitoring plan 34
13 Deviations from clinical investigation plan 34
14 Protocol amendments 34
15 Publication strategy 35
16 Compliance statement 35
16.1 Ethics and Regulatory Considerations ..... **Error! Bookmark not defined.**
16.2 Insurance 35
17 References 36

Etikprövningsmyndigheten
2023-00034-02-350928
2023-01-03

1 Definitions and Abbreviations

AE	Adverse Event
AKI	Acute Kidney Injury
ALAT	Alanine aminotransferase
Alb	Albumin
ASAT	Aspartate aminotransferase
APTT	Activated Partial Thromboplastin Time
CE	Conformité Européene
CNS	Central Nervous System
Crea	Creatinine
CRRT	Continuous Renal Replacement Therapy
ELSO	Extracorporeal Life Support Organization
eGFR	estimated Glomerular Filtration Rate
FiO2	Fraction of inhaled oxygen
g	gram
h	Hour
ICU	Intensive Care Unit
ID	Identification
INR (PT)	International Normalized Ratio (Prothrombin Time)
KDIGO	Kidney Disease: Improving Global Outcome
l	Liters
MA	Marketing Authorisation
mGFR	measured Glomeruli Filtration Rate
min	minute
ml	milliliter
n	nano
U-NAG	Urine - N-acetyl-β-D-glucosaminidase
P	Plasma
PaCO2	Partial pressure of carbon dioxide in artery

Etikprövningsmyndigheten
2023-00034-02-350928
2023-01-03

PaO2	Partial Pressure of Oxygen in Artery
PfHb	Plasma Free Hemoglobin
SAE	Serious Adverse Event
SAP	Statistical Analysis Plan
S	Serum
TNI/TNT/T	Troponin I/T
U	Urin

Etikprövningsmyndigheten
2023-00034-02-350928
2023-01-03

2 Background

The invention of cardiopulmonary bypass/ extracorporeal circulation (ECC) in the 1950's has made open cardiac surgery possible, and more than 200,000 procedures are performed annually in the U.S. alone.[1] However, up to one third of the patients develop postoperative acute kidney injury (AKI), which renders increased morbidity, mortality and costs.[2] The use of ECC may negatively affect the kidneys through several mechanisms, among them impaired renal circulation, increased inflammation and hemolysis, all of which can contribute to renal ischemia and AKI.[3,4] Before the cardiopulmonary bypass machine is connected to the patient, it is filled with 1 – 1.5 L priming fluid, *primed,* usually with crystalloid solution in accordance with the ELSO treatment guidelines.[9] When ECC is initiated, the mixing of blood and priming fluid will lead to hemodilution and reduction of the colloid osmotic (oncotic) pressure. A decrease in oncotic pressure may lead to increased extravasation of fluid and tissue edema, which may impair microcirculation.[5]

PrimECC® is a priming solution for ECC based on Ringers Lactate supplemented with the colloid Dextran 40 and with Dextran 1. In an animal model of ECC, the use of PrimECC® lead to a better maintained oncotic pressure, an improved fluid balance and a reduction of tissue edema compared to a crystalloid priming solution.

In humans, the effect of PrimECC® on oncotic pressure during and after ECC was investigated in a randomized clinical study of 20 + 20 patients in 2005, at Lund University Hospital, Sweden (Widerström M & Steen S, unpublished data). The patients receiving PrimECC® had higher oncotic pressures during ECC compared to patients receiving crystalloid solutions. No side effects were noted, but the study lacked statistical power to evaluate the safety of the new priming solution.

A second randomized, blinded, clinical trial to study the safety and efficacy of PrimECC® was performed at Sahlgrenska University Hospital, Gothenburg, Sweden, during 2016 and 2017.[10] This study included 39 + 41 patients undergoing cardiac surgery with ECC and compared priming with PrimECC® to Ringers acetate with mannitol. Patients with known severe kidney or liver disease, malignancies, sepsis, systemic inflammatory disease or bleeding disorder were excluded. The primary endpoint was oncotic pressure during ECC, with fluid balance and organ function as secondary endpoints. In the PrimECC® group, the oncotic pressure was higher during and early after ECC, and the patients received less intravenous fluids. In addition, the PrimECC-patients had significantly less postoperative renal tubular injury measured as urinary N-acetyl-ß-D-glucoseaminidase (NAG), and reduced levels of hemolysis measured as free plasma-hemoglobin. The study revealed no significant differences in perioperative liver- and lung function, AKI, bleeding, use of blood products or cerebral or myocardial injury markers.

In summary, the use of PrimECC® has been associated with improved perioperative fluid balance, reduced tubular cell injury and reduced hemolysis. However, the studies have been underpowered to detect clinical endpoints such as postoperative AKI and there is no additional published data available on the direct comparison of colloidal and crystalloid priming solutions in ECC for cardiac surgery. The present study is designed to investigate if priming with PrimECC®, compared to crystalloid solution, will lead to a reduced incidence of AKI in a group of elective cardiac surgery patients identified to be at high risk for post-operative kidney injury. As secondary outcomes, incidence of renal replacement therapy, NAG-excretion and estimated glomerular filtration rate (eGFR) will be evaluated. Furthermore, the level of hemolysis and myocardial and CNS injury biomarkers will be assessed.

Etikprövningsmyndigheten
2023-00034-02-350928
2023-01-03

3 Pre-clinical data

Preclinical testing of PrimECC® was done in a porcine model, however, the results were not published due to proprietary reasons. Sixteen pigs of about 60 kgs were randomized to evaluate the differential hemodynamic effects of priming the ECC with PrimECC® versus crystalloid (non-oncotic) solution. Cardiopulmonary bypass was maintained for 60 minutes, and the pigs were monitored for an additional 120 minutes, before sacrifice. The oncotic pressure was maintained and significantly higher in the PrimECC®, group and more fluid were given to the control group to maintain blood pressure.

4 Clinical data

1. The effect of PrimECC® on oncotic pressure during and after cardiac surgery was investigated in a randomized study in 2005, comprising 20 control patients receiving Ringer's acetate and 20 patients receiving PrimECC® as the priming solution (Widerström M & Steen S, unpublished data). The oncotic pressure was maintained during the operation in the PrimECC®-group but was significantly reduced in the crystalloid group. Furthermore, the fluid balance was improved in the PrimECC® group. No side effects were reported.

2. A blinded randomized prospective clinical trial comparing priming with PrimECC® and Ringer's acetate plus mannitol (control group) was performed at Sahlgrenska University Hospital, between May 2016 and June 2017.[10] The primary outcome was oncotic pressure during and after ECC. Patients between 50- and 80-years undergoing elective cardiac surgery with an expected ECC-time >75 minutes were eligible for inclusion. Pre-operative renal or hepatic failure, malignancy, previous cardiac surgery or known bleeding disorders constituted exclusion criteria. In total, 80 patients were included and randomized to PrimECC (n=39) or controls (n=41). The demographics were comparable between the groups.

Etikprövningsmyndigheten
2023-00034-02-350928
2023-01-03

The main findings were that priming with PrimECC® lead to a maintained oncotic pressure during and early after ECC and a significant reduction of perioperative fluid administration compared to controls. In addition, there was a significant reduction in hemolysis and the postoperative NAG-excretion in the PrimECC® group, suggesting a beneficial effect on the kidneys. However, the study was underpowered for detection of group differences in postoperative AKI. There were no significant differences in bleeding or use of blood products. No anaphylactic reactions from PrimECC® were reported.

5 Products to be evaluated

PrimECC® is a physiological salt solution containing dextran 1 and dextran 40. The solution is sterile and intended for single use only. PrimECC® is CE-marked and used according to the certified intended use and in line with local clinical routine.

The XVIVO Click Adapter is to be connected to the green click port connector on the bag.

5.1 Test group

The ECC priming solution PrimECC® (the investigational device) will be used to prime the ECC circuit before the start of subject perfusion in the test group.

5.2 Control group

The standard crystalloid ECC circuit priming solution (the comparator) used at the participating centers i.e., Ringer's acetate with or without mannitol will be used to prime the ECC circuit before the start of subject perfusion in the control group. A mix of two crystalloid priming solutions is allowed. The

standard solution is CE-marked (if classified as medical device) or has a Marketing Authorization and is used according to the certified intended use or within the scope of the Marketing Authorization.

Colloid priming solutions are not allowed in the study but could be administered to the subject as clinically indicated.

5.3 Volume of priming solutions

The ECC priming volume should be according to the standard of care at the respective participating clinic. The typical priming volume is between 1000ml and 1500ml. The same volume of solution must be used for priming the ECC machine in both arms of the study (test group and control group). If additional volume is administered to the subject after priming the ECC machine and during the perfusion of the subject, crystalloid solution should be used and *not* additional PrimECC®.

5.4 Method of priming

Priming of the ECC should be done according to standard of care at the respective participating clinic.

Retrograde autologous priming (RAP) where the subject´s systematic pressure is used to replace the crystalloid solution with autologous blood is not to be used in the study as this method reduces the net priming volume.

5.5 Instruction for use PrimECC®

Etikprövningsmyndigheten
2023-00034-02-350928
2023-01-03

Description

PrimECC® is a CE-marked physiological salt solution containing dextran 1 and dextran 40. The solution is sterile and intended for single use only.

PrimECC® is supplied in bags equipped with a port system with one twist off port for injection and one twist off port with click connector for administration. The material in the bag is a multilayer foil consisting of polypropylene, polyethylene, and polyester. Each bag is sealed in an outer polypropylene/polyamide bag. The XVIVO Click Adapter is to be connected to the green click port connector on the bag.

Intended purpose

PrimECC® is intended as a priming solution for the extracorporeal circulatory (ECC) circuit prior to connecting it to the patient's circulation at onset of cardiopulmonary bypass.

PrimECC® is not intended for any other use than stated above.

Contraindications

Known hypersensitivity to dextran.

Manifest renal injury with oliguria or anuria.

Warning

Do not use PrimECC® if the solution is unclear, the container or outer bag is damaged, or if the 'use by' date has expired.

PrimECC® is intended for single use only and MUST NOT BE REUSED.

Maintain aseptic technique when making any additions as well as during administration.

Do not use the injection port for administration.

Precautions

PrimECC® is ready to use, no additives are necessary.

The responsibility for correct clinical use and technique rests with the user. The Instructions for Use is only provided as a suggestion for procedure, the user must on the basis of her or his medical training and experience evaluate the suitability of this procedure.

PrimECC® must not be used for fluid therapy pre- or postoperatively. The maximum dosage of 1.5 g dextran 40 per kg body weight must not be exceeded (= 33 ml PrimECC®/kg body weight).

PrimECC® contains lactate which may slightly increase plasma lactate levels (by about 6 mmol/l).

Unfractionated heparin should be used cautiously post-operatively as the interaction with dextran might results in excessive bleeding.

Pregnancy: There are no data on the use of PrimECC® during pregnancy. PrimECC® should only be administered to pregnant women if strictly needed.

Lactation: It is not known to what extent PrimECC® passes into breast milk. In the absence of human data, breastfeeding may be continued if clearly needed considering the benefit of breast feeding for the child and the benefit of therapy for the woman.

Etikprövningsmyndigheten
2023-00034-02-350928
2023-01-03

Treatment of children: There are no data on the use of PrimECC® on children. PrimECC® should only be administered to children if strictly needed.

Adverse Effects

Dextran has been associated with rare anaphylactic reactions (<1 in 1000) such as urticaria, fever, chills, pruritus and anaphylaxis.

Any invasive procedure carries a risk of infection.

Instructions

1. Read and understand all the instructions below before proceeding. A standard, sterile transfer set equipped with a spike, a tubing clamp and a XVIVO Click Adapter are needed for an aseptic transfer of PrimECC® to the extracorporeal circulation system. Use aseptic technique throughout the whole procedure.

2. When the outer bag is removed, check that the container is intact by compressing it. Do not use PrimECC® if the solution is unclear, the container or outer bag is damaged, or if the 'use by' date has expired.

3. Instruction on how to connect the bag to the XVIVO Click Adapter.

XVIVO Click Adapter

I. Remove the XVIVO Click Adapter from its sterile packaging.
II. Connect the XVIVO Click Adapter to a tubing set.
III. Clamp the tubing set.
IV. Twist off the closing cap of the green click port on the bag (Picture 1).

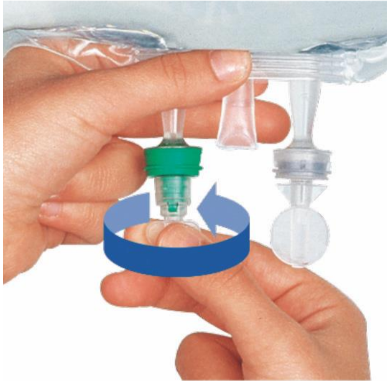

Picture 1

V. Remove the protection cap on the connector of the XVIVO Click Adapter. Push the connector of the XVIVO Click Adapter into the green click port on the bag until it is locked (Picture 2).

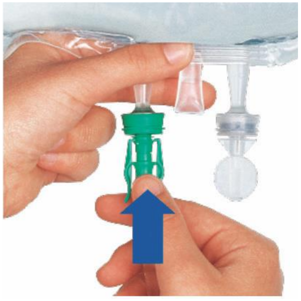

Picture 2

I. For removal of the XVIVO Click Adapter, press the side-wings of the XVIVO Click Adapter and pull out the connector (Picture 3).

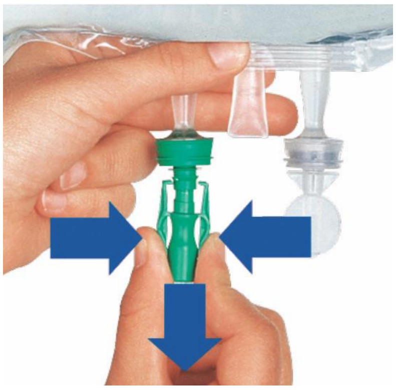

Picture 3

How supplied

PrimECC® is supplied as 6*1600 ml bags and 6*XVIVO Click Adapters per carton.

Store between +2– +25°C.

Symbols

Etikprövningsmyndigheten
2023-00034-02-350928
2023-01-03

STERILE	Sterilized using steam
STERILE EO	Sterilized using ethylene oxide
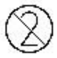	Do not re-use, discard after procedure. Reuse of PrimECC® is not allowed due to the risk for infection.
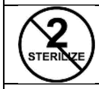	Do not resterilize
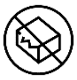	Do not use if the product sterilization barrier or its packaging is compromised.
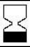	Use by
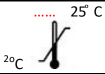	Store between 2–25°C.
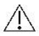	Caution: Consult accompanying documents.
LOT	Batch code
REF	Catalogue number
DEX	Containing dextran 1 and dextran 40
Rx only	Caution: Federal US laws restricts this device to sale by or on the order of a physician.
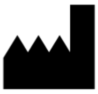	Manufacturer
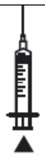	Injection Port
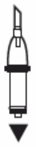	Administration Port

www.xvivoperfusion.com

Manufacturer
XVIVO Perfusion AB
Mässans gata 10
SE-412 51 Göteborg
Sweden

5.6 Product distribution

Five boxes of PrimECC® will be sent out at initiation of the trial at each centre. Each centre will request a reasonable amount of product depending on their expected inclusion rate and storage capability at study initiation. Further deliveries will be made as reasonable and upon request, to complete the total number of patients in the trial. Each box of PrimECC® Product ref No 19054 contains six bags of PrimECC® and six click adaptors. One bag and one click adapter is used for each patient.

Etikprövningsmyndigheten
2023-00034-02-350928
2023-01-03

5.7 Product accountability

XVIVO will provide each study centre with the priming solution PrimECC®. The control group products will be supplied by the study centre in accordance with the current clinical standard at each centre.

The accountability document must be filled in by the person in charge of receiving the study solution at each study centre. The recording method defined and approved during the study initiation visit by the study monitor will be followed throughout the study. Following data are to be recorded for each study product:

- Date of receipt at the study centre
- Batch/lot number
- Expiry date
- Date of use
- Treated patient's study identification
- Date of and reason for return of unused expired or malfunctioning product (if applicable)

6 Study Hypotheses and Objectives

6.1 Hypothesis

The main hypothesis is that, in a population at high risk for postoperative AKI, priming the ECC circuit with PrimECC® instead of only crystalloid solution will lead to a reduced rate of postoperative AKI.

The secondary hypothesis is that priming of the ECC circuit with PrimECC®, compared to a crystalloid solution, will lead to less tubular cell injury, less hemolysis, better fluid balance and better organ preservation.

Etikprövningsmyndigheten
2023-00034-02-350928
2023-01-03

6.2 Primary objective

The primary objective is to evaluate the differential effects of priming with PrimECC® or crystalloid solution on the incidence of post-operative AKI in a high-risk population.

6.3 Secondary objective

The secondary objectives are to study the safety parameters when PrimECC® is used in a large cohort of patients, especially related to bleeding and anaphylactic events, and to assess renal, and myocardial injury.

6.4 Exploratory objective

The exploratory endpoint is to investigate Central nervous system (CNS) injury and NAG/creatinine ratio is a marker to investigate renal tubular impairment.

6.5 Primary endpoint

The primary endpoint is incidence of postoperative AKI of any stage according to the KDIGO creatinine criteria[6] (serum-creatinine increase ≥ 26.5 µmol/l within 48 h or ≥ 50 % increase from baseline) within 96 h after ECC.

6.6 Secondary endpoint

The secondary endpoints are differences between groups in

- eGFR, the highest post-operative value within 96 hours after ECC compared to pre-operative (day before surgery), calculated using the CKD-EPI formula.[7]

- Incidence of AKI of different stages according to the KDIGO creatinine criteria within 96 hours after ECC.[6]
- Hemolysis, after induction of anesthesia and at 1-hour after induction of ECC, 1-hour post- ECC and 24 hours post- ECC., measured as plasma free hemoglobin (PfHb).
- Myocardial injury markers, TNI/TNT, after induction of anesthesia and at 1-hour post- ECC and 24 hours post- ECC. TNI or TNT is chosen according to institutional standard.

6.7 KDIGO creatinine criteria, staging of AKI

Stage	Serum creatinine
1	1.5- 1.9 times baseline OR ≥0.3 mg/dl (≥26.5 µmol/l) increase
2	2.0- 2.9 times baseline
3	3.0 times baseline OR Increase in serum creatinine to ≥4.0 mg/dl (≥353.6 µmol/l) OR, in patients <18 years, decrease in eGFR to <35 ml/min per 1.73m^2

6.8 Exploratory endpoints

The samples that are collected to study exploratory endpoints are optional. The exploratory endpoints are differences between groups in

- CNS injury markers; Tau, NFL, SNE and S100B, after induction of anesthesia, 1-hour post- ECC and 24 hours post- ECC.
- NAG-excretion (U-NAG/U-creatinine) at time-points: after induction of anesthesia, at 1-hour after induction of ECC, at 1-hour post- ECC and 24 hours post- ECC.

Etikprövningsmyndigheten
2023-00034-02-350928
2023-01-03

6.9 Other pre-specified outcome measures

Post-operative bleeding measured as drain loss during first 24 h post operatively, need for transfusions, erythrocytes, plasma, thrombocytes, hemostatic pharmaceuticals, and platelet count, INR and APTT, post-operative lung and liver function as measured through need of respirator at 24 hours, time on ventilator and ALAT/ASAT, respectively.

6.10 Estimated sample size and power

A power analysis was performed using Fisher's test, based on including a population with a 50% risk of any AKI and a presumed reduction of any AKI in the test group to 35%, with 80% power and $\alpha=0.05$. This analysis resulted in a total population of 366 included patients.

7 Study Design

This is a prospective, multi center, double-blinded, randomized controlled clinical trial. The main outcomes will be reported as intention to treat. An as treated analysis will also be performed. This study design is chosen as the trial is considered to be of low risk to the participating patients, based on previous clinical investigations and as prospective randomized trials are considered state-of-the-art. The anticipated risk for participating patients is considered to be comparable in both treatment arms, therefore, the randomization does not lead to an additional burden for the patient. The study has been designed to involve as little pain, discomfort, fear and any other foreseeable risk as possible for the subjects. Patient safety is monitored continuously to identify potential unknown risk that would affect patient safety or alter the risk-benefit assessment in an unfavorable way.

7.1 Risks and Benefits

7.1.1 Anticipated clinical benefits

The document clinical benefit through use of the device PrimECC®, is increased oncotic pressure, improved fluid balance peri-operatively and reduced hemolysis during ECC, compared to when a crystalloid priming solution is used.

7.1.2 Anticipated adverse device effects

Dextran has been associated with rare anaphylactic reactions (<1 in 1000) such as urticaria, fever, chills, pruritus and anaphylaxis.

7.1.3 Risks associated with participation in the clinical investigation

Open cardiac surgery and use of ECC is a high-risk procedure. There are no known additional risks related to participation in this clinical trial. If anaphylaxis would occur, the patient is under strict surveillance at the time and the reaction could be treated.

7.1.4 Risk/benefit assessment

The low risk of anaphylaxis is reasonable considering the positive effect of improved fluid balance and reduced hemolysis compared to when a crystalloid solution is used to prime the ECC device. With the possible additional benefit of reduced AKI that will be investigated in this trial, the benefit side would be even further strengthened.

Etikprövningsmyndigheten
2023-00034-02-350928
2023-01-03

7.2 Study Groups

Patients enrolled according to the inclusion/exclusion criteria will, after written informed consent, be randomized to test group or control group. Randomization will be done through means of a randomization module.

An optimized allocation will be used based on;

eGFR ≥60/eGFR <60

Expected ECC time ≤60 minutes/Expected ECC time >60 minutes

Both groups will receive medical care, based solely on clinical practice and their medical needs.

7.2.1 Blinding procedure

This is a double-blinded study. The only unblinded staff in the study is the perfusionist responsible for the randomization, priming of ECC machine, accountability of the investigational device (PrimECC®), and the dispensing record of the investigational device and comparator. The unblinded perfusionist should in their best effort, hide the identity of the priming solution used, for all the staff performing/ attending the surgery and follow-up care.

Ideally the perfusionist running the ECC during surgery is unaware of the priming solution used and does not adjust perfusion or fluid therapy based on this.

Health care professionals involved in the surgery and follow-up standard of care should not be influenced to act and assess events on the knowledge of what kind of priming solution used.

7.2.2 Emergency unblinding

In case of an emergency where knowledge of the allocated priming solution is required the unblinded perfusionist is responsible for ensuring decoding of the subject.

The unblinded perfusionist is responsible for having the randomization result available for decoding at any time during the study period from Randomization until End of Study visit.

If unblinding is necessary, a Decoding Subject Form should be filled out with information of reason for decoding, description of event and safety event classification, if applicable. The form should be sent to the Sponsor within 24h.

7.3 Study Subjects

A total of 366 subjects aged ≥18 years, , that will undergo elective or urgent (non-emergency) cardiac procedure requiring the use of ECC, with an "Acute kidney injury risk score" ≥30% according to Birnie *et al* 2014,[8] will be included after informed written consent. Subjects that require pre-operative dialysis, have known bleeding disorders, ongoing sepsis or endocarditis, known intolerance or contraindication to Dextran and patients undergoing emergency surgery will be excluded. Antithrombotic medication, except acetylsalicylic acid, should be discontinued before surgery according to the Institutions standard protocol.

<u>Inclusion criteria</u>

- Has signed and dated the EC approved informed consent
- Subject is ≥ 18 years of age
- Requires elective or urgent (non-emergency) cardiac procedure requiring the use of ECC.
- Subject has a pre-operative "Acute Kidney Injury Risk Score" ≥ 30%

  AKI score automatically calculated in eCRF CASTOR for eligible subjects.

  Calculate eGFR (Cockroft Gault Formula) which is used to calculate Acute Kidney Injury Risk Score at https://qxmd.com/calculate/calculator_51/crcl-cockroft-gault

<u>Exclusion criteria</u>

- Unable to give informed consent
- Known bleeding disorder
- Has a known intolerance or contraindication to dextran
- Requires emergency cardiac procedure/surgery
- Currently using an antithrombotic medication which has not been discontinued per institution protocol
- Has ongoing sepsis or endocarditis
- Requires pre-operative dialysis
- The subject is considered by the Investigator to be unsuitable to participate in the investigation for any other reason.

Etikprövningsmyndigheten
2023-00034-02-350928
2023-01-03

7.4 Schedule of assessment

Table 1 Schedule of Assessment

	Pre-operative	After induction of anesthesia	1 h ± 15 min after initiation of ECC	1 h ± 15 min post-ECC	24 ± 3h post- ECC	Postoperative day 2 (within 48h after ECC)	Postoperative day 3 (within 72h after ECC)	Postoperative day 4 (within 96h after ECC)
Informed Consent (up to 1 months)	X							
Assessment/ Evaluation	X							
Medical history and demographics	X							
AKI score[1]	X							
Eligibility review[2]	X							
Randomization	X							
Hemoglobin	X							
PFHb		X	X	X	X			
ASAT, ALAT		X			X			
Serum creatinine[3]	X	X		X	X	X	X	X
Mechanical renal support					X	X	X	X
INR, APTT		X			X			
TNI/TNT		X		X	X			
U-NAG[4], U-Crea, U-Alb		X	X	X	X			
Tau and NFL, NSE and S100B[5]		*X*		*X*	*X*			
Fibrinogen		X			X			
Blood gas		X	X	X	X			
Platelet count		X			X			
Fluid balance		X			X			
Transfusion		X			X	X	X	X
AE/SAE[6]		X	X	X	X	X	X	X

[1] Pre-operative, one day before planned surgery. Surgery postponed ≥ 7days, recalculation of AKI score required.

[2] Pre-operative, one day before planned surgery.

[3] Surgery postponed ≥ 7days, a new s-crea required.

[4] Collection of urine samples for analysis of U-NAG is optional.

[5] Collection of plasma and serum samples for analysis of CNS injury markers is optional.

[6] Evaluation of new AE/SAE until Postoperative day 4. Ongoing AE/SAE will be followed-up until discharge from hospital.

Etikprövningsmyndigheten
2023-00034-02-350928
2023-01-03

7.5 Timeline

It is expected that the study will be performed Q1 2020 to Q2 2024. The subject follow-up period is 96 h after ECC, and the study will be considered completed at 96 hours after ECC of the last included subject.

8 Schedule of Assessments

A preliminary review of medical records should be performed to assess if a subject will be eligible for participation in the study. This includes medical history, renal status, liver status, heart and lung status and any infectious diagnosis. Investigators or delegated staff are responsible for obtaining informed consent and authorization to use and disclose protected health information before randomization. The study does not require specific screening study procedures other than a medical chart review. A final review of the subject´s medications and medical history will be confirmed at time of surgery to make sure that nothing has changed since consenting to the study. The "Acute Kidney injury risk score" will be used to evaluate that the subject fulfil the inclusion criteria at admission the day before surgery. If, for any reason, the surgery is postponed ≥ 7days, a recalculation of AKI score is required with a retaken sample of s-crea.

8.1 Informed Consent

Subjects who are eligible for inclusion will be given written and oral information in advance to allow for time to consider participation*. All subjects participating shall give consent prior to any study activity. A copy of the signed and dated consent shall be provided to the subject.

**Note, only applies in Norway: The investigator or his/her authorized designee obtaining the Informed consent should not be the same that performs the surgical procedure in order to avoid any coercion or undue improper influence on, or inducement of, the subject to participate.*

8.2 Prior to surgery

When a subject who meets eligibility criteria and consents to participate, the center will perform randomization. Subject data, i.e., gender, age, BMI, height, weight, presence of diabetes, kidney function, heart function (LVEF; most recent assessment pre-operative), liver function, hypertension (pharmaceutical treatment or > 140/90 mmHg), smoking history, self- reported ethnicity, Euroscore (optional) and medication is registered as well as type of planned procedure.

8.3 After induction of anesthesia

When the arterial line is set and the subject is anesthetized blood is drawn and analyzed for serum-creatinine, PfHb, ASAT, ALAT, Tau, NFL, NSE, S100B, fibrinogen, platelets, APTT, INR, TNI/TNT and blood gases. *Collection of plasma and serum for analysis of Tau, NFL, NSE, and S100B is optional.*

A urine sample is taken and analyzed for U-NAG (*U-NAG analysis is optional)*, U-creatinine and U-Alb.

8.4 1- hour after induction of ECC

At 1 hour ± 15 minutes after induction of ECC a urine sample is collected to analyze U-NAG (*U-NAG analysis is optional)*, U-Crea and U-Alb. And a blood sample is drawn from arterial line to analyze for PfHB and blood gases.

8.5 1-hour post-ECC

At 1 hour ±15 minutes post- ECC a urine sample is collected to analyze for U-NAG (*U-NAG analysis is optional)*, U-Crea, U-Alb and blood is drawn to analyze serum-creatinine, PfHb, Tau, NFL, NSE, S100B,

Etikprövningsmyndigheten
2023-00034-02-350928
2023-01-03

TNI/TNT and blood gases. *Collection of plasma and serum for analysis of Tau, NFL, NSE, and S100B is optional.*

8.6 24 hours post -ECC

At 24±3 hours post ECC, a urine sample is collected to analyze for U-NAG (*U-NAG analysis is optional)*, U-Crea and U-Alb and blood is drawn to analyze serum-creatinine, PfHb, ASAT, ALAT, Tau, NFL, SNE, S100B, fibrinogen, platelet count, APTT, INR, TNI/TNT and blood gases. *Collection of plasma and serum for analysis of Tau, NFL, NSE, and S100B is optional.*

8.7 Postoperative day 2 (within 48 h after ECC)

Within 48 hours after ECC, blood is drawn to analyze serum-creatinine.

8.8 Postoperative day 3 (within 72 h after ECC)

Within 72 hours after ECC, blood is drawn to analyze serum-creatinine.

8.9 Postoperative day 4 (within 96 h after ECC)

Within 96 hours after ECC, blood is drawn to analyze serum-creatinine.

8.10 Intraoperative parameters

Intraoperative parameters comprising anesthesia start time, ECC time, aortic cross-clamp time, surgery time from start of incision until closure of incision and ventilator time are registered. The subject fluid balance, including all infusions of crystalloids and colloids, transfusions, bleeding and urine output are registered.

8.11 Postoperative parameters

In addition to the laboratory analysis defined above, the patient fluid balance is registered as the sum of infusion of crystalloids, colloids, transfusions, bleeding and urine output. All infusions of blood and/or plasma products are recorded during the study period. Reoperation for bleeding, time on ventilator, significant infections, and ICU length of stay and hospital length of stay are registered.

Use of mechanical renal support such as continuous renal replacement therapy (CRRT) or hemodialysis is registered during the study period.

8.12 Discharge

At discharge, the investigator or delegated staff registers the date and time of discharge and any ongoing adverse events.

8.13 Suspension or termination of the clinical investigation

In case of a safety concern from any of the participating centers that is confirmed by the safety board, the study might be suspended or terminated by the safety board.

9 Adverse events

9.1 Adverse event definition and reporting

An AE is any untoward medical occurrence, unintended disease or injury of any untoward clinical signs (including an abnormal laboratory finding) in subjects, users or other persons, in the context of a clinical investigation, whether or not related to the investigational medical device.

Etikprövningsmyndigheten
2023-00034-02-350928
2023-01-03

This definition includes

a. Events that are anticipated as well as unanticipated.
b. Events occurring in the context of a clinical investigation related to the investigational device, the comparator or the procedures involved.

For the purpose of this protocol, an AE will be defined as any adverse medical change (i.e., de novo or increased severity in a preexisting condition) from the subject's baseline condition, that occurs during the course of the clinical study, whether considered device-related or not and meeting the protocol reporting requirements.

AE reporting requirement applies to both the test group (PrimECC®) and control group (standard crystalloid priming solution). AEs will be collected from the induction of anesthesia until the last subject's last study visit. Ongoing AEs at subject´s last study visit at postoperative day 4, will be followed up until discharge from hospital.

Symptoms caused by the surgical procedure are expected to occur. The following are examples of unavoidable events caused by the surgical procedure are not considered AEs:

- Anesthesia related nausea/vomiting
- Vocal cord dysfunctions
- Low grade fever (less than 38 °C) secondary to chest tube presence.
- Incision pain, redness, and inflammation.
- Shortness of breath not meeting respiratory failure definition
- Post-operative procedure complications
- Mild to moderate bruising at the surgical site
- Back pain related to operating room table
- Sleep issues (insomnia)
- Gastrointestinal discomfort (i.e., constipation, decreased appetite, diarrhea)
- Pulmonary observations (i.e., wheezing, cough)
- Cardiovascular observations (i.e., phlebitis, arrhythmias, cold extremities)
- Neurological observations (i.e., hallucinations, confusion)
- Urological observations (i.e., urinary tract infection, urinary retention)
- There may be additional unavoidable events not listed which can be documented in the source by the investigator.

9.2 AE determination

AE determination is based on three levels of evidence. If level 1 can be determined, then level 2 and 3 events may be updated to the highest level of reporting.

Etikprövningsmyndigheten
2023-00034-02-350928
2023-01-03

Level 1 – final diagnosis

Level 2 – signs

Level 3 – symptoms

Every effort should be made to collect Level 1 evidence of any AE. If an AE has all three levels of evidence, report the AE only once at the highest level of evidence, which is the final diagnosis (Level 1). A single AE should not be reported as multiple AEs based on separate symptoms, signs, and diagnosis.

In any case where a diagnosis is not possible, AE determination should be based on the next highest level of evidence, signs (Level 2), followed by symptoms (Level 3), if only symptoms are available.

A corrective action, itself is not an AE. The AE should always be determined based on the reason that corrective action is taken.

9.3 Serious adverse event

A Serious Adverse Event is one that led to anything of the following:

1. death

2. serious deterioration in the health of the subject, that resulted in any of the following

   - life-threatening illness or injury,
   - permanent impairment of a body structure or a body function,
   - hospitalization or prolongation of hospitalization,
   - medical or surgical intervention to prevent life-threatening illness or injury, or permanent impairment to a body structure or a body function
   - chronic disease
   - fetal distress, fetal death or a congenital physical or mental impairment or birth defect

3. A planned hospitalization for a pre-existing condition or a procedure required by this protocol, without a serious deterioration in health, is not considered to be a SAE.

In the study all AKI related events that are requiring any type of intermittent dialysis or continuous renal replacement therapy (CRRT) should be reported as an AE/SAE.

9.4 Adverse device effect

An adverse device effect (ADE) is an AE that is related to an investigational medical device. This definition includes any AE resulting from insufficiencies or inadequacies in the instructions for use, the composition, the use, or any malfunction of the investigational medical device. It also includes any event that is a result of a user error or intentional misuse.

9.5 Serious adverse device effect

A serious ADE (SADE) is defined as an ADE that has resulted in any of the consequences characteristic of an SAE.

9.6 Unanticipated serious adverse device effect (USADE)

A UADE is any serious adverse effect on health or safety, any life-threatening problem or death caused by, or associated with a device, if that effect, problem, or death was not previously identified in nature,

Etikprövningsmyndigheten
2023-00034-02-350928
2023-01-03

severity, or degree of incidence in the investigational plan (e.g., ICF, Study Protocol, Instructions for Use (IFU), publications, etc.), or any other unanticipated serious problem associated with a device that relates to the rights, safety or welfare of subjects.

9.7 Reporting of all adverse events

The signs, symptoms and sequelae of an underlying AE should not be reported as separate AEs. All reportable AEs must be recorded on the CRF in accordance with the protocol AE reporting requirements. All AEs must also be described by duration (start and resolution dates), severity, relationship to the investigational device, comparator and/or procedure, action taken to resolve the event, outcome of the event, and whether it is considered to be serious or not. Additional information, such as procedural notes, treatment notes, or a signed clinical summary, may be required as supporting documentation for the reported AE.

During the study, all deaths must be reported to the Sponsor within 24 hours of the investigator’s (or a designated staff person’s) knowledge of the death. These also should be reported on the End of Study CRF. A copy of death records, medical records for the events that led to the subject’s death, death certificate (if available) and an autopsy report (if performed) must be sent to the Sponsor as soon as they become available.

9.8 Relationship of the AE to the study device or study procedure

Etikprövningsmyndigheten
2023-00034-02-350928
2023-01-03

The sponsor and the investigator will distinguish between the AE related to the investigational device or comparator and those related to the procedure The procedure in this context is restricted to the pre-operative procedures involving priming of the ECC and the on-going use of ECC during surgery. The procedure does not include the complete surgery and surgical complications not related to the use of ECC should not be assessed for relatedness. Safety events occurring from start of the pre-operative procedures until Postoperative day 4 (96h) could be related to the procedure. The definition of procedure will be the same for both the test group and the control group.

Safety events occurring from start of ECC, when the solution enters the subject´s circulation, and until Postoperative day 4 (96h), could be related to the investigational device and comparator and a relationship assessment should be made. An AE can be related both to procedures and the investigational device.

The relationship between the use of the medical device or the procedure and the occurrence of each AE shall be assessed and categorized. During causality assessment activity, clinical judgement shall be used and the relevant documents, such as the Instructions for Use and the Clinical Investigation Plan shall be consulted, as all the foreseeable serious adverse events and the potential risks are listed and assessed there. The presence of confounding factors, such as concomitant medication/treatment, the natural history of the underlying disease, other concurrent illness or risk factors shall also be considered.

For the purpose of harmonizing reports, each AE will be classified according to four different levels of causality, described below.

1. Not related
2. Possible
3. Probable
4. Causal relationship

The sponsor and the investigators will use the following definitions to assess the relationship of the serious adverse event to the investigational device, the comparator or the investigation procedure.

1. **Not related** - Relationship to the investigational device or procedures can be excluded when

   - the event has no temporal relationship with the use of the investigational device, or the procedures related to application of the investigational device;
   - the serious adverse event does not follow a known response pattern to the medical device (if the response pattern is previously known) and is biologically implausible;
   - the discontinuation of medical device application or the reduction of the level of activation/exposure
   - when clinically feasible - and reintroduction of its use (or increase of the level of activation/exposure), do not impact on the serious adverse event;
   - the event involves a body-site or an organ that cannot be affected by the device or procedure;
   - the serious adverse event can be attributed to another cause (e.g., an underlying or concurrent illness/ clinical condition, an effect of another device, drug, treatment or other risk factors);
   - the event does not depend on a false result given by the investigational device used for diagnosis, when applicable;

In order to establish the non-relatedness, not all the criteria listed above might be met at the same time, depending on the type of device/procedures and the serious adverse event.

2. **Possibly related** - The relationship with the use of the investigational device or comparator, or the relationship with procedures, is weak but cannot be ruled out completely. Alternative causes are also possible (e.g., an underlying or concurrent illness/ clinical condition or/and an effect of another device, drug or treatment). Cases where relatedness cannot be assessed, or no information has been obtained should also be classified as possible.
3. **Probably related** - The relationship with the use of the investigational device or comparator, or the relationship with procedures, seems relevant and/or the event cannot be reasonably explained by another cause.
4. **Causal relationship** - The serious adverse event is associated with the investigational device, comparator or with procedures beyond reasonable doubt when:

   - the event is a known side effect of the product category the device belongs to or of similar devices and procedures;
   - the event has a temporal relationship with investigational device use/application or procedures;
   - the event involves a body-site or organ that the investigational device or procedures are applied to; or the investigational device or procedures have an effect on;
   - the serious adverse event follows a known response pattern to the medical device (if the response pattern is previously known);
   - the discontinuation of medical device application (or reduction of the level of activation/exposure) and reintroduction of its use (or increase of the level of activation/exposure), impact on the serious adverse event (when clinically feasible);

Etikprövningsmyndigheten
2023-00034-02-350928
2023-01-03

- other possible causes (e.g. an underlying or concurrent illness/ clinical condition or/and an effect of another device, drug or treatment) have been adequately ruled out;
- harm to the subject is due to error in use;
- the event depends on a false result given by the investigational device used for diagnosis, when applicable

In order to establish the relatedness, not all the criteria listed above might be met at the same time, depending on the type of device/procedures and the serious adverse event.

9.9 Degree of severity

The degree of severity of the AE to the subject's health will be documented on the AE CRF.

1 = Mild- intervention not indicated or limited intervention

2 = Moderate- non-urgent intervention indicated

3 = Severe- urgent intervention, debilitating, life-threatening, requires or prolongs hospitalization, death.

9.10 Significant infection

Any significant infection is reported between time of 24h post-ECC and postoperative day 4. This is defined as requiring treatment with systemic antibiotics and a positive urine/blood and/or sputum culture. This excludes infections already present at baseline or considered nosocomial.

Etikprövningsmyndigheten
2023-00034-02-350928
2023-01-03

9.11 Device deficiency

A device deficiency is any inadequacy of a medical device with respect to its identity, quality, durability, reliability, safety or performance. This includes malfunctions, use errors and inadequate information supplied by the manufacturer.

9.12 Expedited reporting of serious adverse events and device deficiencies

Any observed or reported SAE (regardless of perceived relationship of the event to the study product) or any observed device deficiency associated with the use of the study products that could have led to an SAE (if suitable action had not been taken, intervention had not been made, or circumstances had been less fortunate), must be reported to XVIVO. Sites are instructed to complete AE report forms in the eCRF and mark the event as "Serious" **within 24 hours** of the investigator or his/her designee becoming aware of the SAE's occurrence. The report should be completed by the investigator or his/her designee. An alert e-mail will automatically be sent to XVIVO. When XVIVO has received the e-mail alert, the Clinical Trial Manager (CTM), or other delegated staff, will send a confirmation receipt to the site **within 24 hours**. If the site does not receive a confirmation receipt from the sponsor within 24 hours, the investigator or his/her designee should e-mail the sponsor to ensure that the SAE has not been overlooked (see contact details below).

If the eCRF cannot be accessed at the time of the occurrence of the SAE or DD, the investigator or his/her designee fills out a paper ***SAE form*** respectively a ***DD form***.

The completed ***SAE form*** should be submitted via e-mail to XVIVO within 24 hours of the investigator or his/her designee becoming aware of the SAE's occurrence. The e-mail should be sent to:

safety.reporting@xvivogroup.com

The completed ***DD form*** should be submitted via e-mail to XVIVO within 24 hours of the investigator or his/her designee becoming aware of the DDs occurrence. The e-mail should be sent to:

satu.hjartstam@xvivogroup.com **and** marina.fredholm@xvivogroup.com

Regardless of the utilized method for SAE reporting, the report should contain (at least) the following information:

I. Record ID (Subject ID)
II. Start date of SAE
III. Stop date of SAE (if known)
IV. Event description
V. Reason AE is considered Serious
VI. Severity
VII. Relationship to Device
VIII. If SAE was treated. If yes, description of treatment
IX. Outcome of SAE
X. Any source data as applicable

N.B. Any patient protected health information site provide must be pseudonymized!

Etikprövningsmyndigheten
2023-00034-02-350928
2023-01-03

Based on local and national requirements, the Investigator or sponsor will also promptly report any such SAE or device deficiency to the responsible EC or regulatory authority in writing and will provide the other party (ie, Investigator or sponsor) with a copy of the notification.

10 Data collection and management

The investigator will ensure that all data collected in the study are recorded in a timely manner according to any instructions provided.

An electronic Case Report Form (eCRF) will be used for data collection. The investigator will ensure that the data are recorded and that any corrections in the CRF as specified in the study protocol and in accordance with the instructions provided. The investigator ensures the accuracy, completeness and timeliness of the data recorded. The investigator will sign the completed CRF. A copy of the completed CRF will be archived at the study site.

All data should be recorded, handled and stored in a way that allows its accurate reporting, interpretation and verification. All source data including informed consent, a copy of the completed CRF, original protocol with amendments and the final report will be stored for a minimum period of ten years after termination of the trial, in accordance with Swedish regulation/law.

Staff designated by/working on behalf of the Sponsor will review the data entered into the CRFs by investigational staff for completeness and accuracy and instruct the site personnel to make any required corrections or additions. Queries are issued electronically. Designated investigator site staff is required to respond to the query and confirm or correct the data.

At the conclusion of the study, the occurrence of any protocol deviations will be determined. After these actions have been completed and the database has been declared to be complete and accurate, it will be locked and available for data analysis.

The investigator must maintain source documents for each subject in the study. A source data verification log will be included in the Investigator Study File (ISF).

10.1 Peri- and postoperative renal function

The renal function is analyzed based on serum creatinine levels, eGFR (calculated by the CKD-EPI formula: https://qxmd.com/calculate/calculator_251/egfr-using-ckd-epi, please note that this formula is not the same as the formula which is used to calculate eGFR to determine the AKI risk score) and AKI (according to the KDIGO creatinine criteria). Tubular injury (NAG-excretion) will be determined with U-NAG/U-Crea.

10.2 Peri- And postoperative liver function

The liver function is measured with ASAT and ALAT.

10.3 Peri- and postoperative pulmonary function

The pulmonary function is assessed as time on ventilator. Ventilation time >24 hours is considered prolonged.

10.4 Peri and post-operative heart injury

The cardiac injury is measured with TNI/TNT (according to the routing use of the centers).

10.5 CNS Injury

CNS injury will be monitored with the CNS biomarkers Tau, NFL, SNE and S100B as an exploratory objective and samples are subject to voluntary collection.

10.6 Peri- and postoperative Hemolysis

Hemolysis will be measured as PfHb.

10.7 ICU Length of stay

Calculated from time patient arrives at the ICU until the patient is discharged from the ICU. In a hospital where there is no step-down unit available to discharge the patient to, there is no bed available, or other unforeseen issues that are not related to the patient's health, but the patient is ready for discharge, then the PI must make a note in the chart that states that the patient was ready to be discharged. This note will be written confirmation of the patient's readiness for discharge and will act as the discharge date and time for patient.

10.8 Hospital length of stay

Calculated from date and time of surgery until date and time that the patient is discharged from the hospital. If the patient must stay in the hospital for another night due to transportation or other unforeseen issues, then the date that the discharge order is written will act as the date of discharge.

11 Statistical analysis plan

Data analysis will be performed by a trained statistician using appropriate statistical methods and software. Descriptive statistics will be presented as means, standard deviations, medians, Q1, Q3 and ranges for the continuous variables and as counts and percent for categorical variables. Mixed models for repeated measures (MMRM) adjusted for baseline will be used for all scale variables that are repeatedly measured. If applicable a robust sandwich estimation will be performed if non-normally distributed data. For comparison between the two randomized groups Fisher's non-parametric permutation test will be used for continuous variables, Fisher's exact test for dichotomous variables,

Etikprövningsmyndigheten
2023-00034-02-350928
2023-01-03

Mantel-Haenszel Chi-square test for ordered categorical variables and Pearson chi-square test for unordered categorical variables. Wherever possible, mean difference between the two groups will be given with 95% confidence interval. All significance tests will be two-sided and conducted at the 5% significance level.

The results from the laboratory analysis will be calculated based on continuous variables. When a clinically relevant cut-off exists also the categorical variable will be used for statistics according to above.

An interim analysis may be performed to evaluate the conditional power to ensure that effect assumptions and study design are valid. Details will be described in the Statistical Analysis Plan (SAP).

11.1 Populations for analysis

The full analysis set (FAS) will consist of all randomized patients. Following the intent-to-treat principle, patients will be analyzed according to the priming solution used (PrimECC and control respectively) to which they were assigned at randomization. Efficacy variables will be analyzed based all randomized patients as the primary population.

The Per Protocol (PP) population will be all subjects in the FAS without any significant protocol violations. In the PP analysis subjects will be analyzed according to their actual treatment.

The FAS population and the PP population will be defined at the clean file meeting before the database lock.

Etikprövningsmyndigheten
2023-00034-02-350928
2023-01-03

11.2 Patient demographics/ other baseline characteristics

Baseline value is defined as the last non-missing assessment prior to surgery. Summary statistics will be provided by treatment group, both for FAS and PP populations, for demographics and baseline characteristics, including age, sex, weight, height, body mass index (BMI), self-reported ethnicity, diabetes, smoking history, dyspnea, hypertension, ejection fraction (LVEF), time of angiography, operative priority, planned cardiac procedure, peripheral vascular disease, triple vessel disease, eGFR, prior hospitalization, and Euroscore (optional). Continuous variables will be summarized using n, mean, standard deviation, median, Q1, Q3, minimum, and maximum. Categorical variables will be summarized using frequency and percentage.

The p-values will be provided for descriptive purposes and will not be considered to define any formal basis for determining factors to be included in statistical models.

11.3 Analysis of the primary variable

The primary analysis of this study is to test if use of the new priming solution reduces AKI compared to when a non-colloid priming solution is used. Any AKI versus no AKI will be compared between the groups using two-sided Fisher's exact test on the FAS population. The mean difference in percentage AKI between the two groups will be given with 95% confidence interval. The relative risk in AKI will be given with 95% confidence interval.

11.4 Analysis of secondary objective

The secondary objective of this study is to test if use of the new priming solution reduces the levels of hemolysis and or alleviate the reduction of eGFR commonly associated with the surgical procedure, compared to when a non-colloid priming solution is used. These repeated continuous variables will be analyzed using mixed models for repeated measures (MMRM) adjusted for baseline on both FAS population and PP-population.

Other secondary objectives include to define safety variables in terms of bleeding, coagulation, pulmonary, cardiac and liver function and to assess fluid balance.

11.5 Analysis of exploratory variable

The exploratory end point of this study is to test if use of the new priming solution reduces the levels of U-NAG/U-Crea and CNS markers (Tau, NFL, SNE and S100B) that are commonly associated with the surgical procedure, compared to when a non-colloid priming solution is used. These repeated continuous variables will be analyzed using mixed models for repeated measures (MMRM) adjusted for baseline on both FAS population and PP-population.

11.6 Safety

The assessment of safety will be based primarily on the frequency of adverse events and laboratory abnormalities and on percentage of subjects with adverse events and laboratory abnormalities. Other safety data will be summarized as appropriate. Laboratory data will be summarized by presenting shift tables using extended normal ranges (baseline to most extreme post-baseline value), by presenting summary statistics of raw data and change from baseline values (mean, medians, standard deviations, ranges).

11.7 Missing Data

All data analyses will be performed on data available. A sensitivity analysis to address missing data will be performed for the primary endpoint in the case of more than 5% missing in either treatment group with multiple imputation. No imputation or corrections will be made for missing data for secondary analyses. Every attempt shall be made to obtain data per protocol. Resources to obtain data include patient or coordinator/physician interview, and chart review. Hence, missing data is expected to be minimal.

Etikprövningsmyndigheten
2023-00034-02-350928
2023-01-03

11.8 Protocol Deviations

A protocol deviation is any event where investigator or study personnel did not conduct the study according to the protocol or applicable laws.

Prior approval from the Principal Investigator is expected if the investigator anticipates a deviation, except when it is necessary to protect the life or physical well-being of a subject in an emergency. Prior approval is not expected when unforeseen circumstances are beyond the investigator's control.

Report all deviations (regardless of cause or prior approval) on the Deviation Form. Notify the sponsor of any deviations within **10 working days**. Notify the EC in accordance with EC policy.

Notify the sponsor within **5 working days** in case of the following deviations:

- Informed consent not obtained prior to surgery or other study activity.
- Incorrect version of consent used.
- Subject failed inclusion/exclusion.
- Source data permanently missing.
- Unauthorized physician performed study-related surgery or procedure.

12 Monitoring plan

A study monitor will be appointed by the sponsor. The monitor will be appropriately trained and informed about the nature of the study, subject written information, ISO 14155:2020 and applicable regulatory requirements. The monitor's qualifications will be documented.

The monitor will have regular contacts with the clinic to verify informed consents of participating subjects, to confirm that facilities remain acceptable, that the investigational team is adhering to the CIP, that data are being accurately recorded in the CRFs, to verify inclusion/exclusion criteria, study main endpoints, check safety reporting and that therapy accountability is being carried out. The investigator should ensure that all persons assisting with the trial are adequately informed and trained about the protocol, the investigational products(s) and their trial related duties and factions. The monitor will check that training has been performed and that this is documented. The monitor will also ensure source data verification (comparison of the data in the CRF with the medical records and other source data). The monitor must have direct access to source data. The extent of monitoring will be defined in a monitoring plan.

13 Deviations from clinical investigation plan

Investigator is not allowed to deviate from the CIP. Under emergency circumstances, deviations from the CIP to protect the rights, safety and well-being of subjects may proceed without prior approval of XVIVO and the EC. Such deviations shall be documented and reported to the sponsor and the EC as soon as possible. Deviations shall be reported to XVIVO to be able to analyze them and assessing their significance.

The reasons for withdrawal and discontinuation of any subject from the investigation shall be recorded. If the discontinuation is because of safety reasons or lack of effectiveness the subject shall still be followed up during the investigation if possible.

14 Protocol amendments

Any change or addition to this protocol requires a written protocol amendment (which can be substantial or not according to regulation) that must be approved by XVIVO before implementation. Amendments affecting the safety of patients, the scope of the investigation or the scientific quality of the study, require additional approval of the EC responsible for each investigator site and in some countries of the Competent Authority. Examples of amendments requiring such approval are:

- A significant change in the study design (e.g., addition or deletion of a control group)
- An increase in the number invasive procedures to which patients are exposed
- Addition or deletion of a test procedure for safety monitoring

XVIVO is responsible for distribution of an amendment to the Competent Authority if applicable, the responsible EC, study investigators and other persons that are involved in the study. Investigators are responsible for the distribution of the amendment to the members of their team.

A copy of the approval of the EC, which becomes part of the protocol, must be provided to XVIVO. If any substantial changes are made to the study design, these approvals must be obtained prior to the enrollment of new patients.

Etikprövningsmyndigheten
2023-00034-02-350928
2023-01-03

15 Publication strategy

The main primary and secondary outcomes will be published as intention to treat and as treated. It is expected that the study will generate a main article in peer reviewed data. The articles are written by the participating centers with the PI as the last author. XVIVO will review the article and might provide suggestions but will not interfere with the publications unless it contains proprietary information that might become subject to patent protection. If that would occur, XVIVO might delay publication for up to three months, until a patent application has been filed.

16 Compliance statement

16.1 Ethics and Regulatory Considerations

The study will be performed in compliance with the protocol, with ethical principles that have their origin in the Declaration of Helsinki and are consistent with ISO 14155:2020 and applicable regulatory requirements.

The study manager must receive a copy of the approval documentation provided by the responsible EC, clearly identifying the protocol version approved, prior to the enrollment of patients in the study and before sending investigational or comparator products and providing access to the CRF. All additional requirements imposed by the EC shall be followed

16.2 Insurance

The patients in the Study are covered by the mandatory subject insurance at the Institution. Concerning the direct or indirect responsibility of the investigational device during this clinical study, XVIVO is liable, on behalf of the Investigators and their assistants for any damage caused to the subject, provided that the Investigators and assistants have complied with XVIVO's instructions specified in this CIP and related amendments, that the investigational device used during this clinical study has been supplied by XVIVO, and that the Investigators and their assistants have conducted this clinical study in accordance with ISO 14155:2020 requirements, scientific practice, techniques and know-how in force.

Etikprövningsmyndigheten
2023-00034-02-350928
2023-01-03

17 References

1. D'Agostino RS, Jacobs JP, Badhwar V, et al. The Society of Thoracic Surgeons Adult Cardiac Surgery Database: 2018 Update on Outcomes and Quality. The Annals of thoracic surgery 2018;105:15-23.
2. Kumar AB, Suneja M. Cardiopulmonary bypass-associated acute kidney injury. Anesthesiology 2011;114:964-70.
3. O'Neal JB, Shaw AD, Billings FTt. Acute kidney injury following cardiac surgery: current understanding and future directions. Critical care 2016;20:187.
4. Vermeulen Windsant IC, de Wit NC, Sertorio JT, et al. Hemolysis during cardiac surgery is associated with increased intravascular nitric oxide consumption and perioperative kidney and intestinal tissue damage. Front Physiol 2014;5:340.
5. Hirleman E, Larson DF. Cardiopulmonary bypass and edema: physiology and pathophysiology. Perfusion 2008;23:311-22.
6. Kellum JA, Lameire N, Group KAGW. Diagnosis, evaluation, and management of acute kidney injury: a KDIGO summary (Part 1). Critical care 2013;17:204-18.
7. Levey AS, Stevens LA, Schmid CH, et al. A new equation to estimate glomerular filtration rate. Ann Intern Med 2009;150:604-12.
8. Birnie K, Verheyden V, Pagano D, et al. Predictive models for kidney disease: improving global outcomes (KDIGO) defined acute kidney injury in UK cardiac surgery. Critical care 2014;18:606.
9. ELSO Guidelines For Adualts Respiratory Failure, version 1.4, August 2017; https://www.elso.org/Resources/Guidelines.aspx
10. Barbu M, Kolsrud O, Ricksten S-E, et al. Dextran_ Versus Crystalloid_ Based Prime in Cardiac Surgery: A Prospective Randomized Pilot Study. Ann Thorac Surg. 2020 Nov;110(5):1541-1547.

Etikprövningsmyndigheten
2023-00034-02-350928
2023-01-03
