# Supplementary material for: Dextran vs. Crystalloid Priming Solution in Cardiac Surgery: A Randomized Trial on Acute Kidney Injury
Source: Acta Anaesthesiol Scand. 2025 Oct 27;70(1):e70139. doi: 10.1111/aas.70139 (PMC12558647; doi:10.1111/aas.70139)
Supplement: Supplementary file 2 — Data S2: Supporting Information. [file AAS-70-0-s001.docx]

| Statistical Report  Project: Priming II  Sponsor: XVIVO Perfusion |
| --- |
| **February 04, 2024**  Authored by:  Aldina Pivodic, aldina.pivodic@apnc.se, +46 709 47 14 12 Annika Wennersten, annika.wennersten@apnc.se, +46 735 40 52 20  Revisions:   \| Nr \| Description \| Date \| \| --- \| --- \| --- \| \| 1 \| First version \| 20231120 \| \| 2 \| Added AKI stages to tables 3.1, and 3.2. Added primary analyses by center for mITT population in table 3.3. \| 20231122 \| \| 3 \| Added height, BMI and euroscore in table 2.1 and 2.2  Corrected AKI grades in table 3.1 to correctly specify missing values for AKI grades instead of zeros, when creatinine is missing.  Corrected ASAT and ALAT in table 7.1 and 7.2. \| 20231123 \| \| 4 \| Added tables 2.3 and 2.4, and variables to 7.1 and 7.2 \| 20240204 \| |

Table of Contents

[Statistical Considerations 3](#_Toc158032105)

[Methods 3](#_Toc158032106)

[Tables 4](#_Toc158032107)

[Table 1. Study populations (All enrolled patients) 4](#_Toc158032108)

[Table 2.1 Demographics, medical history, baseline evaluations, arrhhythmia and randomization strata by randomized treatment group (mITT population) 5](#_Toc158032109)

[Table 2.2 Demographics, medical history, baseline evaluations, arrhhythmia and randomization strata by actual treatment group (PP population) 7](#_Toc158032110)

[Table 2.3 CPB and Clamp Time by randomized treatment group (mITT population) 9](#_Toc158032111)

[Table 2.4 CPB and Clamp Time by actual treatment group (PP population) 10](#_Toc158032112)

[Table 3.1 Analysis of the primary variable and creatinine values used for calculation of the primary variable (mITT population) 11](#_Toc158032113)

[Table 3.2 Analysis of the primary variable and creatinine values used for calculation of the primary variable (PP population) 13](#_Toc158032114)

[Table 3.3 Analysis of the primary variable by center (mITT population) 15](#_Toc158032115)

[Table 4.1 nalysis of the secondary variable eGFR (using CKD-EPI formula, mL/min/1.73m^2) and eGFR values over time (mITT population) 16](#_Toc158032116)

[Table 4.2 Analysis of the secondary variable eGFR (using CKD-EPI formula, mL/min/1.73m^2) and eGFR values over time (PP population) 18](#_Toc158032117)

[Table 5.1 Analysis of the secondary variable hemolysis over time (mITT population) 20](#_Toc158032118)

[Table 5.2 Analysis of the secondary variable hemolysis over time (PP population) 21](#_Toc158032119)

[Table 6.1 Analysis of the exploratory variable hemodialysis over time (mITT population) 22](#_Toc158032120)

[Table 6.2 Analysis of the exploratory variable hemodialysis over time (PP population) 23](#_Toc158032121)

[Table 7.1 Analysis of the inotropic support, fluid balance and selected lab variables (mITT population) 24](#_Toc158032122)

[Table 7.2 Analysis of the inotropic support, fluid balance and selected lab variables (PP population) 28](#_Toc158032123)

[Table 8.1 Overall summary of adverse events by actual treatment group (Safety population) 32](#_Toc158032124)

[Table 8.2 Incidence of adverse events by SOC and PT by actual treatment group (Safety population) 33](#_Toc158032125)

[Table 8.3 Incidence of serious adverse events by SOC and PT by actual treatment group (Safety population) 35](#_Toc158032126)

[Table 8.4 Incidence of adverse events leading to death by SOC and PT by actual treatment group (Safety population) 36](#_Toc158032127)

# Statistical Considerations

## Methods

Descriptively, continuous variables were presented by mean, standard deviation, median, minimum and maximum and categorical variables by counts and percentages.

For test between two groups Fisher’s exact test was used for dichotomous variables, Mantel-Haenszel chi-square trend test for the ordered categorical variables, chi-square test for non-ordered categorical variables, and Mann-Whitney U-test or two-sample t-test for continuous variables depending on the variable distribution.

For the primary variable, effect size was described by odds-ratio (OR) and as risk ratio (RR) with their respective 95% CI. A p-value of 0.05 is considered significant for the confirmatory primary analysis.

All efficacy analyses were presented for the ITT and PP populations. Adverse events were presented for the safety population.

# Tables

# Table 1. Study populations (All enrolled patients)

| **Variable** | **Total N=101** | **Screening failure N=6** | **PrimeECC N=45** | **SOC N=50** |
| --- | --- | --- | --- | --- |
| Center |  |  |  |  |
| SU | 59 (58.4%) | 4 (66.7%) | 27 (60.0%) | 28 (56.0%) |
| CPH | 30 (29.7%) | 2 (33.3%) | 14 (31.1%) | 14 (28.0%) |
| DRE | 12 (11.9%) | 0 (0.0%) | 4 (8.9%) | 8 (16.0%) |
| Randomized population | 95 (94.1%) | 0 (0.0%) | 45 (100.0%) | 50 (100.0%) |
| Safety population | 92 (91.1%) | 0 (0.0%) | 43 (95.6%) | 49 (98.0%) |
| mITT population | 92 (91.1%) | 0 (0.0%) | 43 (95.6%) | 49 (98.0%) |
| Reason for not included in mITT |  |  |  |  |
| Surgery cancelled | 5 | 4 | 0 | 1 |
| Logistic error (no staff available) | 3 | 2 | 1 | 0 |
| Deterioration prior to surgery | 1 | 0 | 1 | 0 |
| PP population | 76 (82.6%) |  | 35 (81.4%) | 41 (83.7%) |
| Reason for not included in PP |  |  |  |  |
| AKI Risk score below 30 | 5 |  | 2 | 3 |
| Creatinine values missing | 8 |  | 3 | 5 |
| Death during or after surgery | 2 |  | 2 | 0 |
| Intervened by SOC instead of PrimeECC | 1 |  | 1 | 0 |
| Actual treatment |  |  |  |  |
| PrimeECC | 42 (45.7%) |  | 42 (97.7%) | 0 (0.0%) |
| SOC | 50 (54.3%) |  | 1 (2.3%) | 49 (100.0%) |
| Reason for end of study |  |  |  |  |
| Study completed | 89 (96.7%) |  | 41 (95.3%) | 48 (98.0%) |
| Death | 3 (3.3%) |  | 2 (4.7%) | 1 (2.0%) |
| Data are presented as mean±standard deviation, median (range) and number of observations, or number (percentage). For test between two groups with respect to dichotomous variables Fisher's exact test was used, for non-ordered categorical variables Chi-square test, for ordered categorical variables Mantel-Haenszel Chi-square trend test, and for continuous variables Mann-Whitney U-test. | | | | |

# Table 2.1 Demographics, medical history, baseline evaluations, arrhhythmia and randomization strata by randomized treatment group (mITT population)

| **Variable** | **PrimeECC N=43** | **SOC N=49** | **p-value** |
| --- | --- | --- | --- |
| ***Demographics*** |  |  |  |
| Sex |  |  | 0.17 |
| Male | 41 (95.3%) | 42 (85.7%) |  |
| Female | 2 (4.7%) | 7 (14.3%) |  |
| Age (years) | 73.3±5.7 74 (59 - 82) n=43 | 72.3±6.3 75 (52 - 82) n=49 | 0.58 |
| Weight (kg) | 90.5±16.6 88 (67 - 140) n=43 | 92.1±19.3 89 (56 - 130) n=49 | 0.68 |
| Height (cm) | 176.3±6.3 177 (160 - 193) n=43 | 176.3±7.5 178 (154 - 190) n=49 | 0.98 |
| Body mass index (kg/m^2) | 29.2±5.2 28.7 (21.6 - 42.3) n=43 | 29.5±5.2 30.3 (18.8 - 38.8) n=49 | 0.74 |
| Smoking history |  |  | 0.86 |
| Never smoker | 12 (27.9%) | 16 (32.7%) |  |
| Current smoker | 5 (11.6%) | 6 (12.2%) |  |
| Former smoker | 26 (60.5%) | 27 (55.1%) |  |
| Race |  |  |  |
| Non-African-American | 43 (100.0%) | 49 (100.0%) |  |
| Euroscore (%) | 2.9±1.4 2.9 (0.9 - 6.0) n=14 | 6.0±7.3 2.2 (1.2 - 26.7) n=15 | 0.78 |
| ***Medical History*** |  |  |  |
| Cardiac procedure |  |  | 0.06 |
| CABG | 18 (41.9%) | 14 (28.6%) |  |
| CABG + Valvular surgery | 16 (37.2%) | 11 (22.4%) |  |
| Valvular surgery (one valve) | 5 (11.6%) | 16 (32.7%) |  |
| Valvular surgery (two valves) | 2 (4.7%) | 1 (2.0%) |  |
| Aorta surgery | 1 (2.3%) | 1 (2.0%) |  |
| Other | 1 (2.3%) | 6 (12.2%) |  |
| Operative priority |  |  | 0.09 |
| Elective | 23 (53.5%) | 35 (71.4%) |  |
| Urgent | 20 (46.5%) | 14 (28.6%) |  |
| Angio (catheter) to surgery time |  |  | 0.08 |
| >24 hours, this admission | 15 (34.9%) | 9 (18.8%) |  |
| >24 hours, previous admission | 28 (65.1%) | 39 (81.3%) |  |
| Missing | 0 | 1 |  |
| Diabetes |  |  | 0.67 |
| No history of diabetes | 21 (48.8%) | 28 (57.1%) |  |
| Type I | 1 (2.3%) | 1 (2.0%) |  |
| Type II, insulin dependent | 7 (16.3%) | 4 (8.2%) |  |
| Type II, oral treatment | 14 (32.6%) | 16 (32.7%) |  |
| Peripheral vascular disease | 7 (16.3%) | 9 (18.4%) | 1.00 |
| Hypertension | 41 (95.3%) | 48 (98.0%) | 0.60 |
| Triple vessel disease | 22 (51.2%) | 24 (49.0%) | 1.00 |
| ***Baseline Evaluations*** |  |  |  |
| Dyspnea grade |  |  | 0.36 |
| NYHA 1 | 6 (14.0%) | 4 (8.2%) |  |
| NYHA 2 | 16 (37.2%) | 17 (34.7%) |  |
| NYHA 3 | 20 (46.5%) | 27 (55.1%) |  |
| NYHA 4 | 1 (2.3%) | 1 (2.0%) |  |
| Ejecation fraction (%) | 50.1±11.2 52.0 (20.0 - 65.0) n=43 | 48.3±13.9 55.0 (10.0 - 70.0) n=49 | 0.68 |
| Hemoglobin (g/dL) | 13.3±1.8 13.5 (9.3 - 16.8) n=39 | 13.4±1.7 13.7 (9.3 - 17.5) n=45 | 0.63 |
| S-creatinine (µmol/L) | 107.8±33.8 105.0 (51.0 - 192.0) n=43 | 105.1±24.5 101.0 (53.0 - 154.0) n=49 | 0.67 |
| eGFR (using CKD-EPI formula, mL/min/1.73m²) | 63.0±22.1 60.2 (27.4 - 110.1) n=43 | 61.7±18.5 58.8 (30.8 - 114.8) n=49 | 0.85 |
| eGFR (using CrCl Cockroft-Gault formula, mL/min) | 77.4±35.8 74.8 (33.4 - 195.2) n=43 | 77.6±34.7 68.2 (28.4 - 190.1) n=49 | 0.85 |
| Calculated acute kidney risk score (%) | 38.4±9.4 35.6 (27.6 - 70.2) n=43 | 40.1±11.1 35.4 (24.5 - 73.3) n=49 | 0.54 |
| ***Arrhythmia*** |  |  |  |
| Cardiac rhythm |  |  | 0.47 |
| Sinus rhythm | 31 (72.1%) | 38 (77.6%) |  |
| Atrial fibrillation | 12 (27.9%) | 10 (20.4%) |  |
| None of the above | 0 (0.0%) | 1 (2.0%) |  |
| ***Randomization Strata*** |  |  |  |
| eGFR (using CKD-EPI formula) cat. |  |  | 0.30 |
| ≥60 | 25 (58.1%) | 23 (46.9%) |  |
| <60 | 18 (41.9%) | 26 (53.1%) |  |
| Expected time on ECC (min) |  |  | 1.00 |
| ≤60 | 15 (34.9%) | 18 (36.7%) |  |
| >60 | 28 (65.1%) | 31 (63.3%) |  |
| Data are presented as mean±standard deviation, median (range) and number of observations, or number (percentage). For test between two groups with respect to dichotomous variables Fisher's exact test was used, for non-ordered categorical variables Chi-square test, for ordered categorical variables Mantel-Haenszel Chi-square trend test, and for continuous variables Mann-Whitney U-test. | | | |

# Table 2.2 Demographics, medical history, baseline evaluations, arrhhythmia and randomization strata by actual treatment group (PP population)

| **Variable** | **PrimeECC N=35** | **SOC N=41** | **p-value** |
| --- | --- | --- | --- |
| ***Demographics*** |  |  |  |
| Sex |  |  | 0.17 |
| Male | 33 (94.3%) | 34 (82.9%) |  |
| Female | 2 (5.7%) | 7 (17.1%) |  |
| Age (years) | 73.5±5.5 74 (59 - 82) n=35 | 72.1±6.6 73 (52 - 82) n=41 | 0.44 |
| Weight (kg) | 92.0±16.8 91 (67 - 140) n=35 | 90.1±18.8 87 (56 - 127) n=41 | 0.65 |
| Height (cm) | 175.4±5.9 177 (160 - 183) n=35 | 175.1±7.6 176 (154 - 189) n=41 | 0.87 |
| Body mass index (kg/m^2) | 29.9±5.3 30.1 (22.3 - 42.3) n=35 | 29.3±5.3 30.3 (18.8 - 37.9) n=41 | 0.58 |
| Smoking history |  |  | 0.44 |
| Never smoker | 9 (25.7%) | 15 (36.6%) |  |
| Current smoker | 3 (8.6%) | 5 (12.2%) |  |
| Former smoker | 23 (65.7%) | 21 (51.2%) |  |
| Race |  |  |  |
| Non-African-American | 35 (100.0%) | 41 (100.0%) |  |
| Euroscore (%) | 3.1±1.5 3.2 (0.9 - 6.0) n=9 | 6.9±8.3 2.1 (1.2 - 26.7) n=11 | 1.00 |
| ***Medical History*** |  |  |  |
| Cardiac procedure |  |  | 0.032 |
| CABG | 16 (45.7%) | 10 (24.4%) |  |
| CABG + Valvular surgery | 12 (34.3%) | 10 (24.4%) |  |
| Valvular surgery (one valve) | 4 (11.4%) | 15 (36.6%) |  |
| Valvular surgery (two valves) | 2 (5.7%) | 1 (2.4%) |  |
| Other | 1 (2.9%) | 5 (12.2%) |  |
| Operative priority |  |  | 0.24 |
| Elective | 20 (57.1%) | 29 (70.7%) |  |
| Urgent | 15 (42.9%) | 12 (29.3%) |  |
| Angio (catheter) to surgery time |  |  | 0.14 |
| >24 hours, this admission | 11 (31.4%) | 7 (17.1%) |  |
| >24 hours, previous admission | 24 (68.6%) | 34 (82.9%) |  |
| Diabetes |  |  | 0.62 |
| No history of diabetes | 17 (48.6%) | 22 (53.7%) |  |
| Type I | 1 (2.9%) | 1 (2.4%) |  |
| Type II, insulin dependent | 6 (17.1%) | 3 (7.3%) |  |
| Type II, oral treatment | 11 (31.4%) | 15 (36.6%) |  |
| Peripheral vascular disease | 7 (20.0%) | 9 (22.0%) | 1.00 |
| Hypertension | 33 (94.3%) | 40 (97.6%) | 0.59 |
| Triple vessel disease | 18 (51.4%) | 19 (46.3%) | 0.82 |
| ***Baseline Evaluations*** |  |  |  |
| Dyspnea grade |  |  | 0.32 |
| NYHA 1 | 4 (11.4%) | 3 (7.3%) |  |
| NYHA 2 | 14 (40.0%) | 13 (31.7%) |  |
| NYHA 3 | 16 (45.7%) | 24 (58.5%) |  |
| NYHA 4 | 1 (2.9%) | 1 (2.4%) |  |
| Ejecation fraction (%) | 50.4±10.8 55.0 (20.0 - 65.0) n=35 | 47.5±13.7 50.0 (10.0 - 65.0) n=41 | 0.43 |
| Hemoglobin (g/dL) | 13.3±1.6 13.5 (9.3 - 16.8) n=32 | 13.3±1.7 13.7 (9.3 - 17.2) n=37 | 0.74 |
| S-creatinine (µmol/L) | 110.5±34.6 108.0 (51.0 - 192.0) n=35 | 104.2±25.5 100.0 (53.0 - 154.0) n=41 | 0.37 |
| eGFR (using CKD-EPI formula, mL/min/1.73m²) | 61.0±22.2 54.8 (27.4 - 110.1) n=35 | 62.2±19.4 61.5 (30.8 - 114.8) n=41 | 0.74 |
| eGFR (using CrCl Cockroft-Gault formula, mL/min) | 77.3±38.4 66.2 (33.8 - 195.2) n=35 | 77.3±36.3 65.1 (28.4 - 190.1) n=41 | 0.75 |
| Calculated acute kidney risk score (%) | 39.3±9.9 35.6 (30.1 - 70.2) n=35 | 41.8±11.3 36.9 (30.0 - 73.3) n=41 | 0.36 |
| ***Arrhythmia*** |  |  |  |
| Cardiac rhythm |  |  | 0.62 |
| Sinus rhythm | 27 (77.1%) | 32 (78.0%) |  |
| Atrial fibrillation | 8 (22.9%) | 8 (19.5%) |  |
| None of the above | 0 (0.0%) | 1 (2.4%) |  |
| ***Randomization Strata*** |  |  |  |
| eGFR (using CKD-EPI formula) cat. |  |  | 1.00 |
| ≥60 | 18 (51.4%) | 21 (51.2%) |  |
| <60 | 17 (48.6%) | 20 (48.8%) |  |
| Expected time on ECC (min) |  |  | 0.64 |
| ≤60 | 14 (40.0%) | 14 (34.1%) |  |
| >60 | 21 (60.0%) | 27 (65.9%) |  |
| Data are presented as mean±standard deviation, median (range) and number of observations, or number (percentage). For test between two groups with respect to dichotomous variables Fisher's exact test was used, for non-ordered categorical variables Chi-square test, for ordered categorical variables Mantel-Haenszel Chi-square trend test, and for continuous variables Mann-Whitney U-test. | | | |

# Table 2.3 CPB and Clamp Time by randomized treatment group (mITT population)

| **Variable** | **PrimeECC N=43** | **SOC N=49** | **p-value** |
| --- | --- | --- | --- |
| CPB time (hours) | 2.04±1.23 1.8 (0.8 - 7.3) n=43 | 1.80±1.22 1.4 (0.9 - 8.4) n=49 | 0.09 |
| Clamp time (hours) | 1.45±0.75 1.3 (0.6 - 4.5) n=43 | 1.24±0.61 1.1 (0.5 - 4.2) n=48 | 0.10 |
| Data are presented as mean±standard deviation, median (range) and number of observations, or number (percentage). For test between two groups with respect to continuous variables Mann-Whitney U-test was used. | | | |

# Table 2.4 CPB and Clamp Time by actual treatment group (PP population)

| **Variable** | **PrimeECC N=35** | **SOC N=41** | **p-value** |
| --- | --- | --- | --- |
| CPB time (hours) | 1.81±0.92 1.6 (0.8 - 5.7) n=35 | 1.64±0.77 1.4 (0.9 - 4.4) n=41 | 0.26 |
| Clamp time (hours) | 1.35±0.73 1.2 (0.6 - 4.5) n=35 | 1.23±0.64 1.1 (0.5 - 4.2) n=40 | 0.39 |
| Data are presented as mean±standard deviation, median (range) and number of observations, or number (percentage). For test between two groups with respect to continuous variables Mann-Whitney U-test was used. | | | |

# Table 3.1 Analysis of the primary variable and creatinine values used for calculation of the primary variable (mITT population)

| **Variable** | **PrimeECC N=43** | **SOC N=49** | **p-value** |
| --- | --- | --- | --- |
| ***Primary variable*** |  |  |  |
| Acute kidney injury | 35 (83.3%) | 26 (54.2%) | 0.0036 OR:4.23 (1.57 - 11.39) RR:1.54 (1.15 - 2.06) |
| ***AKI stages*** |  |  |  |
| AKI stages at 60 min after removal of ECC |  |  | 0.0024 |
| No AKI | 30 (73.2%) | 47 (95.9%) |  |
| AKI stage 1 | 11 (26.8%) | 2 (4.1%) |  |
| Missing | 2 | 0 |  |
| AKI stages at 0-24h post surgery |  |  | 0.0085 |
| No AKI | 10 (23.8%) | 30 (62.5%) |  |
| AKI stage 1 | 27 (64.3%) | 14 (29.2%) |  |
| AKI stage 2 | 3 (7.1%) | 2 (4.2%) |  |
| AKI stage 3 | 2 (4.8%) | 2 (4.2%) |  |
| Missing | 1 | 1 |  |
| AKI stages at 24-48h post surgery |  |  | 0.0098 |
| No AKI | 11 (26.8%) | 27 (57.4%) |  |
| AKI stage 1 | 20 (48.8%) | 15 (31.9%) |  |
| AKI stage 2 | 7 (17.1%) | 3 (6.4%) |  |
| AKI stage 3 | 3 (7.3%) | 2 (4.3%) |  |
| Missing | 2 | 2 |  |
| AKI stages at 48-72h post surgery |  |  | 0.024 |
| No AKI | 11 (28.9%) | 23 (51.1%) |  |
| AKI stage 1 | 17 (44.7%) | 16 (35.6%) |  |
| AKI stage 2 | 6 (15.8%) | 5 (11.1%) |  |
| AKI stage 3 | 4 (10.5%) | 1 (2.2%) |  |
| Missing | 5 | 4 |  |
| AKI stages at 72-96h post surgery |  |  | 0.16 |
| No AKI | 20 (48.8%) | 26 (56.5%) |  |
| AKI stage 1 | 14 (34.1%) | 18 (39.1%) |  |
| AKI stage 2 | 5 (12.2%) | 1 (2.2%) |  |
| AKI stage 3 | 2 (4.9%) | 1 (2.2%) |  |
| Missing | 2 | 3 |  |
| ***S-Creatinine (µmol/L)*** |  |  |  |
| During induction of anesthesia | 98.8±30.7 96 (52 - 173) n=43 | 94.8±23.4 93 (49 - 151) n=49 | 0.78 |
| 60 min after removal of ECC | 117.4±31.0 115 (66 - 189) n=41 | 100.9±25.6 100 (48 - 161) n=49 | 0.020 |
| During 0-24h post surgery | 152.1±54.3 144 (73 - 302) n=41 | 124.9±41.8 119 (59 - 231) n=48 | 0.015 |
| During 24-48h post surgery | 168.3±67.3 163 (63 - 354) n=41 | 132.5±51.8 120 (59 - 313) n=47 | 0.010 |
| During 48-72h post surgery | 165.0±72.4 148 (63 - 410) n=38 | 133.8±54.4 120 (63 - 344) n=45 | 0.027 |
| During 72-96h post surgery | 143.3±73.3 127 (60 - 436) n=41 | 128.1±55.4 113 (61 - 342) n=46 | 0.25 |
| ***Change in s-creatinine (µmol/L) from induction of anesthesia*** |  |  |  |
| 60 min after removal of ECC | 17.7±12.7 13 (-2 - 53) n=41 | 6.1±12.5 5 (-33 - 45) n=49 | <.0001 |
| During 0-24h post surgery | 53.7±34.6 42 (9 - 133) n=41 | 30.5±34.6 19 (-11 - 153) n=48 | 0.0002 |
| During 24-48h post surgery | 69.9±50.9 69 (0 - 198) n=41 | 38.5±45.1 20 (-22 - 194) n=47 | 0.0015 |
| During 48-72h post surgery | 64.4±59.1 39 (5 - 241) n=38 | 38.9±48.1 22 (-14 - 225) n=45 | 0.017 |
| During 72-96h post surgery | 44.9±55.7 26 (-13 - 267) n=41 | 34.2±50.1 22 (-33 - 223) n=46 | 0.32 |
| ***Percent change in s-creatinine (µmol/L) from induction of anesthesia*** |  |  |  |
| 60 min after removal of ECC | 20.5±18.4 12 (-2 - 79) n=41 | 7.2±14.8 5 (-29 - 60) n=49 | <.0001 |
| During 0-24h post surgery | 57.0±41.6 45 (10 - 235) n=41 | 33.9±39.0 20 (-12 - 196) n=48 | 0.0005 |
| During 24-48h post surgery | 74.6±67.7 61 (0 - 381) n=41 | 42.3±47.1 26 (-18 - 214) n=47 | 0.0043 |
| During 48-72h post surgery | 68.0±66.9 44 (5 - 283) n=38 | 42.8±47.9 36 (-12 - 189) n=45 | 0.054 |
| During 72-96h post surgery | 44.4±46.5 29 (-17 - 158) n=41 | 38.4±53.2 25 (-27 - 283) n=46 | 0.54 |
| Data are presented as mean±standard deviation, median (range) and number of observations, or number (percentage). For test between two groups with respect to dichotomous variables Fisher's exact test was used, for ordered categorical variables Mantel-Haenszel Chi-square trend test was used, and for continuous variables Mann-Whitney U-test. | | | |

# Table 3.2 Analysis of the primary variable and creatinine values used for calculation of the primary variable (PP population)

| **Variable** | **PrimeECC N=35** | **SOC N=41** | **p-value** |
| --- | --- | --- | --- |
| ***Primary variable*** |  |  |  |
| Acute kidney injury | 30 (85.7%) | 22 (53.7%) | 0.0032 OR:5.18 (1.68 - 16.01) RR:1.60 (1.17 - 2.19) |
| ***AKI stages*** |  |  |  |
| AKI stages at 60 min after removal of ECC |  |  | 0.0064 |
| No AKI | 27 (77.1%) | 40 (97.6%) |  |
| AKI stage 1 | 8 (22.9%) | 1 (2.4%) |  |
| AKI stages at 0-24h post surgery |  |  | 0.0083 |
| No AKI | 7 (20.0%) | 26 (63.4%) |  |
| AKI stage 1 | 24 (68.6%) | 12 (29.3%) |  |
| AKI stage 2 | 3 (8.6%) | 1 (2.4%) |  |
| AKI stage 3 | 1 (2.9%) | 2 (4.9%) |  |
| AKI stages at 24-48h post surgery |  |  | 0.0081 |
| No AKI | 7 (20.0%) | 23 (56.1%) |  |
| AKI stage 1 | 19 (54.3%) | 13 (31.7%) |  |
| AKI stage 2 | 6 (17.1%) | 3 (7.3%) |  |
| AKI stage 3 | 3 (8.6%) | 2 (4.9%) |  |
| AKI stages at 48-72h post surgery |  |  | 0.048 |
| No AKI | 10 (28.6%) | 20 (48.8%) |  |
| AKI stage 1 | 16 (45.7%) | 15 (36.6%) |  |
| AKI stage 2 | 5 (14.3%) | 5 (12.2%) |  |
| AKI stage 3 | 4 (11.4%) | 1 (2.4%) |  |
| AKI stages at 72-96h post surgery |  |  | 0.15 |
| No AKI | 16 (45.7%) | 23 (56.1%) |  |
| AKI stage 1 | 13 (37.1%) | 16 (39.0%) |  |
| AKI stage 2 | 4 (11.4%) | 1 (2.4%) |  |
| AKI stage 3 | 2 (5.7%) | 1 (2.4%) |  |
| ***S-Creatinine (µmol/L)*** |  |  |  |
| During induction of anesthesia | 102.5±31.9 103 (52 - 173) n=35 | 92.8±23.4 87 (49 - 151) n=41 | 0.23 |
| 60 min after removal of ECC | 119.5±32.0 115 (66 - 189) n=35 | 97.5±26.0 98 (48 - 161) n=41 | 0.0035 |
| During 0-24h post surgery | 160.0±53.9 147 (75 - 302) n=35 | 123.8±44.2 119 (59 - 231) n=41 | 0.0022 |
| During 24-48h post surgery | 177.8±64.8 163 (74 - 354) n=35 | 133.6±54.4 120 (59 - 313) n=41 | 0.0023 |
| During 48-72h post surgery | 166.0±73.6 145 (63 - 410) n=35 | 134.1±56.7 120 (63 - 344) n=41 | 0.028 |
| During 72-96h post surgery | 149.3±75.7 130 (61 - 436) n=35 | 128.2±57.9 113 (61 - 342) n=41 | 0.10 |
| ***Change in s-creatinine (µmol/L) from induction of anesthesia*** |  |  |  |
| 60 min after removal of ECC | 17.0±13.3 12 (-2 - 53) n=35 | 4.7±11.8 3 (-33 - 36) n=41 | 0.0001 |
| During 0-24h post surgery | 57.5±35.1 46 (9 - 133) n=35 | 31.0±35.5 20 (-11 - 153) n=41 | 0.0002 |
| During 24-48h post surgery | 75.3±49.8 69 (12 - 198) n=35 | 40.8±47.6 20 (-22 - 194) n=41 | 0.0008 |
| During 48-72h post surgery | 63.5±59.8 37 (5 - 241) n=35 | 41.4±49.4 27 (-14 - 225) n=41 | 0.045 |
| During 72-96h post surgery | 46.9±58.0 27 (-13 - 267) n=35 | 35.5±52.5 21 (-33 - 223) n=41 | 0.29 |
| ***Percent change in s-creatinine (µmol/L) from induction of anesthesia*** |  |  |  |
| 60 min after removal of ECC | 19.4±19.3 12 (-2 - 79) n=35 | 5.6±13.4 3 (-29 - 46) n=41 | 0.0004 |
| During 0-24h post surgery | 59.8±43.1 47 (10 - 235) n=35 | 34.2±39.2 20 (-12 - 196) n=41 | 0.0006 |
| During 24-48h post surgery | 79.4±68.3 63 (12 - 381) n=35 | 45.1±49.3 28 (-18 - 214) n=41 | 0.0030 |
| During 48-72h post surgery | 66.0±66.6 40 (5 - 283) n=35 | 45.7±48.9 38 (-12 - 189) n=41 | 0.16 |
| During 72-96h post surgery | 44.8±45.9 29 (-17 - 158) n=35 | 40.1±55.8 25 (-27 - 283) n=41 | 0.56 |
| Data are presented as mean±standard deviation, median (range) and number of observations, or number (percentage). For test between two groups with respect to dichotomous variables Fisher's exact test was used, and for continuous variables Mann-Whitney U-test. | | | |

# Table 3.3 Analysis of the primary variable by center (mITT population)

| **Variable** | **PrimeECC N=43** | **SOC N=49** | **p-value** |
| --- | --- | --- | --- |
| ***Site: SU*** |  |  |  |
| Acute kidney injury | 22 (81.5%) | 11 (39.3%) | 0.0022 OR:6.80 (1.98 - 23.31) RR:2.07 (1.27 - 3.40) |
| ***Site: CPH*** |  |  |  |
| Acute kidney injury | 10 (83.3%) | 9 (75.0%) | 1.00 OR:1.67 (0.22 - 12.35) RR:1.11 (0.74 - 1.68) |
| ***Site: DRE*** |  |  |  |
| Acute kidney injury | 3 (100.0%) | 6 (75.0%) | 1.00 OR: non-est RR:1.11 (0.74 - 1.68) |
| Data are presented as number (percentage). OR = Odds ratio; RR = risk ratio; non-est = non-estimable effect. For test between two groups with respect to dichotomous variables Fisher's exact test was used. | | | |

# Table 4.1 nalysis of the secondary variable eGFR (using CKD-EPI formula, mL/min/1.73m^2) and eGFR values over time (mITT population)

| **Variable** | **PrimeECC N=43** | **SOC N=49** | **p-value** |
| --- | --- | --- | --- |
| ***Secondary variable*** |  |  |  |
| Change in maximum eGFR (using CKD-EPI formula, mL/min/1.73m²) from pre-surgery to post-surgery | -8.8±18.7 -7 (-56 - 43) n=43 | 1.0±20.0 -1 (-60 - 40) n=49 | 0.021 |
| ***eGFR (using CKD-EPI formula, mL/min/1.73m²)*** |  |  |  |
| Pre-surgery | 63.0±22.1 60 (27 - 110) n=43 | 61.7±18.5 59 (31 - 115) n=49 | 0.85 |
| During induction of anesthesia | 66.4±18.8 66 (31 - 95) n=43 | 67.6±17.0 68 (34 - 108) n=49 | 0.92 |
| 60 min after removal of ECC | 57.5±17.8 56 (28 - 89) n=43 | 63.7±17.0 61 (28 - 101) n=49 | 0.13 |
| During 0-24h post surgery | 46.3±20.3 40 (16 - 88) n=43 | 53.7±20.4 51 (20 - 100) n=49 | 0.054 |
| During 24-48h post surgery | 43.4±22.7 36 (13 - 88) n=43 | 52.8±21.6 52 (16 - 100) n=49 | 0.031 |
| During 48-72h post surgery | 47.3±23.5 42 (11 - 91) n=43 | 53.8±22.0 53 (15 - 98) n=49 | 0.13 |
| During 72-96h post surgery | 51.5±22.6 48 (10 - 89) n=43 | 55.6±21.4 55 (15 - 98) n=49 | 0.32 |
| ***Change in eGFR (using CKD-EPI formula, mL/min/1.73m²) from pre-surgery*** |  |  |  |
| During induction of anesthesia | 3.4±8.1 4 (-17 - 25) n=43 | 5.9±9.0 5 (-7 - 38) n=49 | 0.20 |
| 60 min after removal of ECC | -5.5±14.2 -4 (-39 - 38) n=43 | 2.0±13.5 1 (-34 - 38) n=49 | 0.014 |
| During 0-24h post surgery | -16.8±20.6 -15 (-72 - 43) n=43 | -8.0±17.9 -8 (-63 - 32) n=49 | 0.0071 |
| During 24-48h post surgery | -19.6±23.6 -18 (-84 - 43) n=43 | -8.9±19.3 -5 (-64 - 32) n=49 | 0.0053 |
| During 48-72h post surgery | -15.8±23.8 -13 (-82 - 43) n=43 | -7.9±19.6 -4 (-60 - 32) n=49 | 0.051 |
| During 72-96h post surgery | -11.5±20.9 -10 (-71 - 43) n=43 | -6.1±21.4 -6 (-69 - 40) n=49 | 0.34 |
| ***Percent change in eGFR (using CKD-EPI formula, mL/min/1.73m²) from pre-surgery*** |  |  |  |
| During induction of anesthesia | 8.1±14.2 5 (-15 - 51) n=43 | 11.9±18.4 10 (-15 - 88) n=49 | 0.39 |
| 60 min after removal of ECC | -5.6±20.6 -7 (-37 - 76) n=43 | 6.5±25.9 1 (-41 - 93) n=49 | 0.018 |
| During 0-24h post surgery | -23.8±33.4 -29 (-68 - 115) n=43 | -10.7±28.0 -13 (-73 - 56) n=49 | 0.0028 |
| During 24-48h post surgery | -28.8±36.2 -38 (-79 - 115) n=43 | -12.4±30.8 -9 (-75 - 56) n=49 | 0.0027 |
| During 48-72h post surgery | -22.7±35.8 -22 (-77 - 115) n=43 | -10.9±32.4 -7 (-70 - 58) n=49 | 0.022 |
| During 72-96h post surgery | -16.1±34.3 -20 (-69 - 115) n=43 | -6.3±37.7 -12 (-81 - 94) n=49 | 0.22 |
| Data are presented as mean±standard deviation, median (range) and number of observations. For test between two groups Mann-Whitney U-test was used for continuous variables. | | | |

# Table 4.2 Analysis of the secondary variable eGFR (using CKD-EPI formula, mL/min/1.73m^2) and eGFR values over time (PP population)

| **Variable** | **PrimeECC N=35** | **SOC N=41** | **p-value** |
| --- | --- | --- | --- |
| ***Secondary variable*** |  |  |  |
| Change in maximum eGFR (using CKD-EPI formula, mL/min/1.73m²) from pre-surgery to post-surgery | -11.6±14.9 -9 (-56 - 19) n=35 | -2.6±18.8 -3 (-60 - 40) n=41 | 0.053 |
| ***eGFR (using CKD-EPI formula, mL/min/1.73m²)*** |  |  |  |
| Pre-surgery | 61.0±22.2 55 (27 - 110) n=35 | 62.2±19.4 61 (31 - 115) n=41 | 0.74 |
| During induction of anesthesia | 64.1±19.1 63 (31 - 95) n=35 | 68.7±17.5 73 (34 - 108) n=41 | 0.45 |
| 60 min after removal of ECC | 54.9±17.1 54 (28 - 89) n=35 | 65.8±17.6 70 (28 - 101) n=41 | 0.016 |
| During 0-24h post surgery | 41.1±16.8 38 (16 - 80) n=35 | 54.0±21.2 51 (20 - 100) n=41 | 0.0062 |
| During 24-48h post surgery | 37.4±17.4 34 (13 - 80) n=35 | 51.5±22.2 50 (16 - 100) n=41 | 0.0064 |
| During 48-72h post surgery | 41.7±18.9 39 (11 - 82) n=35 | 51.4±21.7 50 (15 - 98) n=41 | 0.054 |
| During 72-96h post surgery | 47.3±20.5 44 (10 - 87) n=35 | 54.1±21.5 54 (15 - 98) n=41 | 0.14 |
| ***Change in eGFR (using CKD-EPI formula, mL/min/1.73m²) from pre-surgery*** |  |  |  |
| During induction of anesthesia | 3.0±7.9 4 (-17 - 25) n=35 | 6.5±9.3 5 (-7 - 38) n=41 | 0.12 |
| 60 min after removal of ECC | -6.1±12.5 -3 (-39 - 16) n=35 | 3.6±13.0 1 (-32 - 38) n=41 | 0.0049 |
| During 0-24h post surgery | -20.0±16.8 -15 (-72 - 17) n=35 | -8.2±16.7 -8 (-63 - 20) n=41 | 0.0029 |
| During 24-48h post surgery | -23.6±19.4 -19 (-84 - 14) n=35 | -10.8±18.5 -7 (-64 - 19) n=41 | 0.0028 |
| During 48-72h post surgery | -19.3±20.1 -13 (-82 - 11) n=35 | -10.9±18.6 -8 (-60 - 22) n=41 | 0.06 |
| During 72-96h post surgery | -13.8±17.2 -11 (-71 - 19) n=35 | -8.1±20.3 -9 (-69 - 40) n=41 | 0.37 |
| ***Percent change in eGFR (using CKD-EPI formula, mL/min/1.73m²) from pre-surgery*** |  |  |  |
| During induction of anesthesia | 7.5±13.4 6 (-15 - 51) n=35 | 13.0±19.1 10 (-10 - 88) n=41 | 0.27 |
| 60 min after removal of ECC | -7.1±16.4 -6 (-37 - 33) n=35 | 9.2±26.2 2 (-37 - 93) n=41 | 0.0057 |
| During 0-24h post surgery | -31.0±20.1 -35 (-68 - 34) n=35 | -11.6±26.5 -13 (-73 - 46) n=41 | 0.0006 |
| During 24-48h post surgery | -37.2±21.2 -41 (-79 - 28) n=35 | -15.6±29.3 -11 (-75 - 50) n=41 | 0.0013 |
| During 48-72h post surgery | -30.2±22.6 -23 (-77 - 22) n=35 | -15.6±30.2 -12 (-70 - 53) n=41 | 0.020 |
| During 72-96h post surgery | -21.8±23.1 -21 (-69 - 39) n=35 | -9.9±35.4 -15 (-81 - 94) n=41 | 0.19 |
| Data are presented as mean±standard deviation, median (range) and number of observations. For test between two groups Mann-Whitney U-test was used for continuous variables. | | | |

# Table 5.1 Analysis of the secondary variable hemolysis over time (mITT population)

| **Variable** | **PrimeECC N=43** | **SOC N=49** | **p-value** |
| --- | --- | --- | --- |
| ***During induction of anesthesia*** |  |  |  |
| Plasma-free hemoglobin <0.1 g/L or 3µmol/L | 9 (20.9%) | 15 (30.6%) | 0.35 |
| Plasma-free hemoglobin (g/L) during induction of anesthesia | 0.18±0.15 0.12 (0.05 - 0.89) n=34 | 0.17±0.08 0.20 (0.05 - 0.40) n=34 | 0.86 |
| ***60 min after induction of ECC*** |  |  |  |
| Plasma-free hemoglobin <0.1 g/L or 3µmol/L | 3 (7.0%) | 1 (2.1%) | 0.34 |
| Plasma-free Hemoglobin (g/L) 60 min after induction of ECC | 0.18±0.09 0.20 (0.00 - 0.40) n=40 | 0.34±0.23 0.27 (0.05 - 1.00) n=47 | 0.0007 |
| ***60 min after removal of ECC*** |  |  |  |
| Plasma-free hemoglobin <0.1 g/L or 3µmol/L | 1 (2.4%) | 1 (2.0%) | 1.00 |
| Plasma-free Hemoglobin (g/L) 60 min after removal of ECC | 0.37±0.22 0.32 (0.08 - 0.95) n=40 | 0.54±0.56 0.40 (0.01 - 3.82) n=48 | 0.09 |
| ***During 0-24h post surgery*** |  |  |  |
| Plasma-free hemoglobin <0.1 g/L or 3µmol/L | 21 (51.2%) | 21 (46.7%) | 0.83 |
| Plasma-free Hemoglobin (g/L) during 0-24h post surgery | 0.10±0.03 0.10 (0.05 - 0.20) n=20 | 0.16±0.13 0.10 (0.05 - 0.70) n=24 | 0.044 |
| Data are presented as mean±standard deviation, median (range) and number of observations, or number (percentage). For test between two groups Fisher's exact test was used for dichotomous variables, and Mann-Whitney U-test for continuous variables. | | | |

# Table 5.2 Analysis of the secondary variable hemolysis over time (PP population)

| **Variable** | **PrimeECC N=35** | **SOC N=41** | **p-value** |
| --- | --- | --- | --- |
| ***During induction of anesthesia*** |  |  |  |
| Plasma-free hemoglobin <0.1 g/L or 3µmol/L | 7 (20.0%) | 15 (36.6%) | 0.13 |
| Plasma-free hemoglobin (g/L) during induction of anesthesia | 0.16±0.09 0.10 (0.06 - 0.40) n=28 | 0.18±0.08 0.20 (0.07 - 0.40) n=26 | 0.39 |
| ***60 min after induction of ECC*** |  |  |  |
| Plasma-free hemoglobin <0.1 g/L or 3µmol/L | 2 (5.7%) | 1 (2.4%) | 0.59 |
| Plasma-free Hemoglobin (g/L) 60 min after induction of ECC | 0.19±0.09 0.20 (0.00 - 0.40) n=33 | 0.34±0.23 0.29 (0.05 - 1.00) n=40 | 0.0029 |
| ***60 min after removal of ECC*** |  |  |  |
| Plasma-free hemoglobin <0.1 g/L or 3µmol/L | 1 (2.9%) | 1 (2.4%) | 1.00 |
| Plasma-free Hemoglobin (g/L) 60 min after removal of ECC | 0.34±0.19 0.30 (0.08 - 0.90) n=34 | 0.47±0.31 0.40 (0.01 - 1.30) n=40 | 0.08 |
| ***During 0-24h post surgery*** |  |  |  |
| Plasma-free hemoglobin <0.1 g/L or 3µmol/L | 18 (51.4%) | 19 (50.0%) | 1.00 |
| Plasma-free Hemoglobin (g/L) during 0-24h post surgery | 0.11±0.02 0.10 (0.10 - 0.20) n=17 | 0.16±0.14 0.10 (0.05 - 0.70) n=19 | 0.15 |
| Data are presented as mean±standard deviation, median (range) and number of observations, or number (percentage). For test between two groups Fisher's exact test was used for dichotomous variables, and Mann-Whitney U-test for continuous variables. | | | |

# Table 6.1 Analysis of the exploratory variable hemodialysis over time (mITT population)

| **Variable** | **PrimeECC N=43** | **SOC N=49** | **p-value** |
| --- | --- | --- | --- |
| ***Exploratory variable*** |  |  |  |
| Any hemodialysis during post-surgery | 3 (7.1%) | 2 (4.2%) | 0.66 |
| ***During 0-24h post surgery*** |  |  |  |
| Hemodialysis | 1 (2.4%) | 2 (4.2%) | 1.00 |
| Type of hemodialysis |  |  |  |
| Continuous renal replacement therapy | 1 | 2 |  |
| ***During 24-48h post surgery*** |  |  |  |
| Hemodialysis | 1 (2.4%) | 2 (4.2%) | 1.00 |
| Type of hemodialysis |  |  |  |
| Continuous renal replacement therapy | 1 | 2 |  |
| ***During 48-72h post surgery*** |  |  |  |
| Hemodialysis | 2 (4.9%) | 1 (2.1%) | 0.59 |
| Type of hemodialysis |  |  |  |
| Continuous renal replacement therapy | 2 | 1 |  |
| ***During 72-96h post surgery*** |  |  |  |
| Hemodialysis | 2 (5.0%) | 1 (2.1%) | 0.59 |
| Type of hemodialysis |  |  |  |
| Intermittent hemodialysis | 0 | 1 |  |
| Continuous renal replacement therapy | 2 | 0 |  |
| ***At discharge*** |  |  |  |
| Hemodialysis | 1 (2.4%) | 0 (0.0%) | 0.46 |
| Type of hemodialysis |  |  |  |
| Intermittent hemodialysis | 1 | 0 |  |
| Data are presented as number (percentage). For test between two groups Fisher's exact test was used for dichotomous variables. | | | |

# Table 6.2 Analysis of the exploratory variable hemodialysis over time (PP population)

| **Variable** | **PrimeECC N=35** | **SOC N=41** | **p-value** |
| --- | --- | --- | --- |
| ***Exploratory variable*** |  |  |  |
| Hemodialysis | 2 (5.7%) | 2 (4.9%) | 1.00 |
| ***During 0-24h post surgery*** |  |  |  |
| Hemodialysis | 0 (0.0%) | 2 (4.9%) | 0.50 |
| Type of hemodialysis |  |  |  |
| Continuous renal replacement therapy | 0 | 2 |  |
| ***During 24-48h post surgery*** |  |  |  |
| Hemodialysis | 1 (2.9%) | 2 (4.9%) | 1.00 |
| Type of hemodialysis |  |  |  |
| Continuous renal replacement therapy | 1 | 2 |  |
| ***During 48-72h post surgery*** |  |  |  |
| Hemodialysis | 2 (5.7%) | 1 (2.4%) | 0.59 |
| Type of hemodialysis |  |  |  |
| Continuous renal replacement therapy | 2 | 1 |  |
| ***During 72-96h post surgery*** |  |  |  |
| Hemodialysis | 2 (5.9%) | 1 (2.4%) | 0.59 |
| Type of hemodialysis |  |  |  |
| Intermittent hemodialysis | 0 | 1 |  |
| Continuous renal replacement therapy | 2 | 0 |  |
| ***At discharge*** |  |  |  |
| Hemodialysis | 1 (2.9%) | 0 (0.0%) | 0.46 |
| Type of hemodialysis |  |  |  |
| Intermittent hemodialysis | 1 | 0 |  |
| Data are presented as number (percentage). For test between two groups Fisher's exact test was used for dichotomous variables. | | | |

# Table 7.1 Analysis of the inotropic support, fluid balance and selected lab variables (mITT population)

| **Variable** | **PrimeECC N=43** | **SOC N=49** | **p-value** |
| --- | --- | --- | --- |
| Inotropes given during ECC | 5 (11.6%) | 5 (10.2%) | 1.00 |
| Vasopressors given during ECC | 18 (41.9%) | 16 (32.7%) | 0.39 |
| Inotropes given during discontinuation of ECC | 8 (18.6%) | 7 (14.3%) | 0.59 |
| Vasopressors given during discontinuation of ECC | 17 (39.5%) | 16 (32.7%) | 0.52 |
| Diuretics given during ECC | 1 (2.3%) | 2 (4.2%) | 1.00 |
| Diuretics given after ECC, before ICU arrival | 11 (25.6%) | 7 (14.6%) | 0.20 |
| Priming solution (mL) during surgery | 1309.3±97.1 1300.0 (1200.0 - 1600.0) n=43 | 1354.2±226.9 1300.0 (800.0 - 2500.0) n=48 | 0.32 |
| Crystalloids (mL) during surgery | 1611.4±1080.1 1223.0 (0.0 - 4500.0) n=43 | 1847.4±1159.6 1527.0 (0.0 - 4528.0) n=48 | 0.33 |
| Colloids (mL) during surgery | 9.3±42.6 0.0 (0.0 - 200.0) n=43 | 96.7±583.3 0.0 (0.0 - 4040.0) n=48 | 0.48 |
| Erythrocytes (mL) during surgery | 178.6±376.3 0.0 (0.0 - 2000.0) n=43 | 84.3±203.8 0.0 (0.0 - 750.0) n=48 | 0.13 |
| Erythrocytes given during surgery | 13 (30.2%) | 8 (16.7%) | 0.14 |
| Erythrocytes from Cellsaver (mL) during surgery | 126.8±331.3 0.0 (0.0 - 1750.0) n=43 | 229.6±412.1 0.0 (0.0 - 1800.0) n=48 | 0.12 |
| Plasma (mL) during surgery | 94.0±390.1 0.0 (0.0 - 2500.0) n=43 | 30.2±176.2 0.0 (0.0 - 1200.0) n=48 | 0.11 |
| Plasma given during surgery | 6 (14.0%) | 2 (4.2%) | 0.14 |
| Thrombocytes (mL) during surgery | 102.8±286.6 0.0 (0.0 - 1600.0) n=43 | 47.5±213.1 0.0 (0.0 - 1100.0) n=48 | 0.08 |
| Thrombocytes given during surgery | 8 (18.6%) | 3 (6.3%) | 0.11 |
| Fibrinogen (g) during surgery | 1.0±1.7 0.0 (0.0 - 6.0) n=20 | 1.2±1.7 0.0 (0.0 - 4.0) n=13 | 0.60 |
| Fibrinogen given during surgery | 6 (30.0%) | 5 (38.5%) | 0.71 |
| Factor II, VII, IX and X (Ocplex, Confidex) (IU) during surgery | 100.0±347.9 0.0 (0.0 - 1500.0) n=20 | 615.4±1063.7 0.0 (0.0 - 3000.0) n=13 | 0.12 |
| Factor II, VII, IX and X (Ocplex, Confidex) given during surgery | 2 (10.0%) | 4 (30.8%) | 0.18 |
| Factor VII concentrate (mg) during surgery | 0.0±0.0 0.0 (0.0 - 0.0) n=20 | 0.0±0.0 0.0 (0.0 - 0.0) n=13 | 1.00 |
| Factor VII concentrate given during surgery | 0(0.0%) | 0(0.0%) |  |
| Bleeding (mL) during surgery | 938.7±1930.5 500.0 (100.0 - 12800.0) n=43 | 659.4±761.0 325.0 (100.0 - 3070.0) n=48 | 0.30 |
| Urine production (mL) during surgery | 483.3±352.4 390.0 (30.0 - 1900.0) n=43 | 509.5±283.9 425.0 (30.0 - 1207.0) n=48 | 0.30 |
| Hemofiltration during ECC (mL) during surgery | 494.0±990.3 0.0 (0.0 - 4840.0) n=43 | 129.2±473.1 0.0 (0.0 - 2600.0) n=48 | 0.0096 |
| Other fluids lost (mL) during surgery | 226.7±293.0 200.0 (0.0 - 1800.0) n=43 | 174.4±177.1 200.0 (0.0 - 900.0) n=48 | 0.27 |
| Net fluid balance (mL) during surgery | 1289.6±1565.6 1270.0 (-1711.0 - 6406.0) n=43 | 2217.3±1658.8 1852.5 (-1917.0 - 6331.0) n=48 | 0.0059 |
| ASAT (µkat/L) during induction of anesthesia | 0.4±0.2 0.4 (0.2 - 1.0) n=42 | 0.5±0.2 0.4 (0.0 - 1.3) n=49 | 0.53 |
| ALAT (µkat/L) during induction of anesthesia | 0.4±0.2 0.4 (0.1 - 1.2) n=42 | 0.5±0.5 0.4 (0.1 - 3.2) n=49 | 0.41 |
| Inotropes given during 0-24h post surgery | 2 (4.8%) | 4 (8.3%) | 0.68 |
| Vasopressors given during 0-24h post surgery | 19 (45.2%) | 20 (41.7%) | 0.83 |
| Diuretics given during 0-24h post surgery | 38 (92.7%) | 42 (87.5%) | 0.50 |
| Crystalloids (mL) during 0-24h post surgery | 3839.3±1645.4 4276.0 (600.0 - 6768.0) n=41 | 4005.4±2258.5 3548.5 (0.0 - 8600.0) n=48 | 0.92 |
| Colloids (mL) during 0-24h post surgery | 198.8±271.2 100.0 (0.0 - 1100.0) n=41 | 129.2±194.6 0.0 (0.0 - 1000.0) n=48 | 0.24 |
| Erythrocytes (mL) during 0-24h post surgery | 244.7±394.7 0.0 (0.0 - 1998.0) n=41 | 132.5±233.1 0.0 (0.0 - 1061.0) n=48 | 0.15 |
| Erythrocytes given post surgery | 18 (43.9%) | 15 (31.3%) | 0.27 |
| Plasma (mL) during 0-24h post surgery | 171.1±487.3 0.0 (0.0 - 2250.0) n=41 | 115.5±420.4 0.0 (0.0 - 2400.0) n=48 | 0.37 |
| Plasma given post surgery | 7 (17.1%) | 5 (10.4%) | 0.54 |
| Thrombocytes (mL) during 0-24h post surgery | 41.4±146.3 0.0 (0.0 - 750.0) n=41 | 34.9±125.4 0.0 (0.0 - 586.0) n=48 | 0.84 |
| Thrombocytes given post surgery | 4 (9.8%) | 4 (8.3%) | 1.00 |
| Fibrinogen (g) during 0-24h post surgery | 1.0±1.3 0.0 (0.0 - 5.0) n=20 | 0.1±0.5 0.0 (0.0 - 2.0) n=17 | 0.015 |
| Fibrinogen given during 0-24h post surgery | 9 (45.0%) | 1 (5.9%) | 0.010 |
| Factor II, VII, IX and X (Ocplex, Confidex) (IU) during 0-24h post surgery | 80.0±244.1 0.0 (0.0 - 1000.0) n=20 | 58.8±242.5 0.0 (0.0 - 1000.0) n=17 | 0.43 |
| Factor II, VII, IX and X (Ocplex, Confidex) given during 0-24h post surgery | 3 (15.0%) | 1 (5.9%) | 0.61 |
| Factor VII concentrate (mg) during 0-24h post surgery | 0.0±0.0 0.0 (0.0 - 0.0) n=20 | 0.0±0.0 0.0 (0.0 - 0.0) n=17 |  |
| Factor VII concentrate given during 0-24h post surgery | 0(0.0%) | 0(0.0%) |  |
| Bleeding (mL) during 0-24h post surgery | 873.3±533.1 750.0 (275.0 - 3030.0) n=41 | 854.2±655.2 565.0 (225.0 - 2900.0) n=48 | 0.14 |
| Urine production (mL) during 0-24h post surgery | 2324.4±819.1 2250.0 (895.0 - 4420.0) n=41 | 2584.8±941.7 2365.0 (1270.0 - 5050.0) n=48 | 0.25 |
| Other fluids lost (mL) during 0-24h post surgery | 190.9±311.6 0.0 (0.0 - 799.0) n=41 | 169.5±307.6 0.0 (0.0 - 916.0) n=48 | 0.67 |
| Net fluid balance (mL) during 0-24h post surgery | 1106.7±1594.2 1115.0 (-3683.0 - 4374.0) n=41 | 809.1±1993.0 801.0 (-5420.0 - 5060.0) n=48 | 0.35 |
| ASAT (µkat/L) during 0-24h post surgery | 1.4±1.2 1.1 (0.6 - 6.8) n=41 | 1.1±0.6 1.0 (0.4 - 2.9) n=48 | 0.07 |
| ALAT (µkat/L) during 0-24h post surgery | 0.5±0.2 0.4 (0.1 - 1.2) n=41 | 0.6±0.5 0.4 (0.2 - 2.8) n=48 | 0.74 |
| INR during 0-24h post surgery (24±3h post ECC) | 1.3±0.3 1.3 (1.0 - 2.8) n=38 | 1.3±0.2 1.3 (1.0 - 1.6) n=45 | 0.34 |
| APTT (s) during 0-24h post surgery (24±3h post ECC) | 28.6±4.7 28.0 (24.0 - 50.0) n=37 | 28.1±3.0 28.0 (23.0 - 41.0) n=44 | 0.89 |
| Fibrinogen (g/L) during 0-24h post surgery (24±3h post ECC) | 4.1±0.4 4.1 (3.3 - 5.1) n=37 | 3.9±0.8 4.0 (1.8 - 6.4) n=44 | 0.24 |
| Thrombocytes (10^9/L) during 0-24h post surgery (24±3h post ECC) | 173.6±69.0 148.0 (62.0 - 421.0) n=41 | 159.9±49.3 151.0 (92.0 - 311.0) n=48 | 0.42 |
| Inotropes given during 24-48h post surgery | 2 (4.9%) | 3 (6.3%) | 1.00 |
| Vasopressors given during 24-48h post surgery | 3 (7.3%) | 5 (10.4%) | 0.72 |
| Fibrinogen (g) during 24-48h post surgery | 0.0±0.0 0.0 (0.0 - 0.0) n=12 | 0.0±0.0 0.0 (0.0 - 0.0) n=15 |  |
| Fibrinogen given during 24-48h post surgery | 0(0.0%) | 0(0.0%) |  |
| Factor II, VII, IX and X (Ocplex, Confidex) (IU) during 24-48h post surgery | 0.0±0.0 0.0 (0.0 - 0.0) n=12 | 0.0±0.0 0.0 (0.0 - 0.0) n=15 |  |
| Factor II, VII, IX and X (Ocplex, Confidex) given during 24-48h post surgery | 0(0.0%) | 0(0.0%) |  |
| Factor VII concentrate (mg) during 24-48h post surgery | 0.0±0.0 0.0 (0.0 - 0.0) n=12 | 0.0±0.0 0.0 (0.0 - 0.0) n=15 |  |
| Factor VII concentrate given during 24-48h post surgery | 0(0.0%) | 0(0.0%) |  |
| Inotropes given during 48-72h post surgery | 2 (4.9%) | 2 (4.2%) |  |
| Vasopressors given during 48-72h post surgery | 2 (4.9%) | 2 (4.2%) |  |
| Fibrinogen (g) during 48-72h post surgery | 0.0±0.0 0.0 (0.0 - 0.0) n=9 | 0.0±0.0 0.0 (0.0 - 0.0) n=7 |  |
| Fibrinogen given during 48-72h post surgery | 0(0.0%) | 0(0.0%) |  |
| Factor II, VII, IX and X (Ocplex, Confidex) (IU) during 48-72h post surgery | 0.0±0.0 0.0 (0.0 - 0.0) n=9 | 0.0±0.0 0.0 (0.0 - 0.0) n=7 |  |
| Factor II, VII, IX and X (Ocplex, Confidex) given during 48-72h post surgery | 0(0.0%) | 0(0.0%) |  |
| Factor VII concentrate (mg) during 48-72h post surgery | 0.0±0.0 0.0 (0.0 - 0.0) n=9 | 0.0±0.0 0.0 (0.0 - 0.0) n=7 |  |
| Factor VII concentrate given during 48-72h post surgery | 0(0.0%) | 0(0.0%) |  |
| Inotropes given during 72-96h post surgery | 1 (2.4%) | 0 (0.0%) | 0.46 |
| Vasopressors given during 72-96h post surgery | 0 (0.0%) | 1 (2.1%) | 1.00 |
| Fibrinogen (g) during 0-24h post surgery | 1.0±1.3 0.0 (0.0 - 5.0) n=20 | 0.1±0.5 0.0 (0.0 - 2.0) n=17 | 0.015 |
| Fibrinogen given during 72-96h post surgery | 0(0.0%) | 0(0.0%) |  |
| Factor II, VII, IX and X (Ocplex, Confidex) (IU) during 72-96h post surgery | 0.0±0.0 0.0 (0.0 - 0.0) n=4 | 0.0±0.0 0.0 (0.0 - 0.0) n=4 |  |
| Factor II, VII, IX and X (Ocplex, Confidex) given during 72-96h post surgery | 0(0.0%) | 0(0.0%) |  |
| Factor VII concentrate (mg) during 72-96h post surgery | 0.0±0.0 0.0 (0.0 - 0.0) n=4 | 0.0±0.0 0.0 (0.0 - 0.0) n=4 |  |
| Factor VII concentrate given during 72-96h post surgery | 0(0.0%) | 0(0.0%) |  |
| Data are presented as mean±standard deviation, median (range) and number of observations, or number (percentage). For test between two groups Fisher's exact test was used for dichotomous variables, and Mann-Whitney U-test for continuous variables. | | | |

# Table 7.2 Analysis of the inotropic support, fluid balance and selected lab variables (PP population)

| **Variable** | **PrimeECC N=35** | **SOC N=41** | **p-value** |
| --- | --- | --- | --- |
| Inotropes given during ECC | 3 (8.6%) | 2 (4.9%) | 0.66 |
| Vasopressors given during ECC | 16 (45.7%) | 14 (34.1%) | 0.35 |
| Inotropes given during discontinuation of ECC | 6 (17.1%) | 7 (17.1%) | 1.00 |
| Vasopressors given during discontinuation of ECC | 15 (42.9%) | 15 (36.6%) | 0.64 |
| Diuretics given during ECC | 1 (2.9%) | 1 (2.4%) | 1.00 |
| Diuretics given after ECC, before ICU arrival | 8 (22.9%) | 7 (17.1%) | 0.57 |
| Priming solution (mL) during surgery | 1308.6±101.1 1300.0 (1200.0 - 1600.0) n=35 | 1365.9±242.5 1300.0 (800.0 - 2500.0) n=41 | 0.18 |
| Crystalloids (mL) during surgery | 1581.7±1061.5 1200.0 (0.0 - 4500.0) n=35 | 1937.3±1196.0 1534.0 (0.0 - 4528.0) n=41 | 0.20 |
| Colloids (mL) during surgery | 5.7±33.8 0.0 (0.0 - 200.0) n=35 | 113.2±630.8 0.0 (0.0 - 4040.0) n=41 | 0.23 |
| Erythrocytes (mL) during surgery | 140.9±246.4 0.0 (0.0 - 980.0) n=35 | 98.7±217.6 0.0 (0.0 - 750.0) n=41 | 0.27 |
| Erythrocytes given during surgery | 11 (31.4%) | 8 (19.5%) | 0.29 |
| Erythrocytes from Cellsaver (mL) during surgery | 92.0±224.1 0.0 (0.0 - 806.0) n=35 | 268.8±434.4 0.0 (0.0 - 1800.0) n=41 | 0.036 |
| Plasma (mL) during surgery | 36.3±108.7 0.0 (0.0 - 500.0) n=35 | 35.4±190.5 0.0 (0.0 - 1200.0) n=41 | 0.31 |
| Plasma given during surgery | 4 (11.4%) | 2 (4.9%) | 0.41 |
| Thrombocytes (mL) during surgery | 70.3±175.0 0.0 (0.0 - 720.0) n=35 | 55.6±230.0 0.0 (0.0 - 1100.0) n=41 | 0.22 |
| Thrombocytes given during surgery | 6 (17.1%) | 3 (7.3%) | 0.29 |
| Fibrinogen (g) during surgery | 1.1±1.8 0.0 (0.0 - 6.0) n=16 | 1.2±1.7 0.0 (0.0 - 4.0) n=13 | 0.72 |
| Fibrinogen given during surgery | 5 (31.3%) | 5 (38.5%) | 0.71 |
| Factor II, VII, IX and X (Ocplex, Confidex) (IU) during surgery | 125.0±387.3 0.0 (0.0 - 1500.0) n=16 | 615.4±1063.7 0.0 (0.0 - 3000.0) n=13 | 0.20 |
| Factor II, VII, IX and X (Ocplex, Confidex) given during surgery | 2 (12.5%) | 4 (30.8%) | 0.36 |
| Factor VII concentrate (mg) during surgery | 0.0±0.0 0.0 (0.0 - 0.0) n=16 | 0.0±0.0 0.0 (0.0 - 0.0) n=13 | 1.00 |
| Factor VII concentrate given during surgery | 0(0.0%) | 0(0.0%) |  |
| Bleeding (mL) during surgery | 664.7±593.7 400.0 (100.0 - 2600.0) n=35 | 676.8±812.8 300.0 (100.0 - 3070.0) n=41 | 0.49 |
| Urine production (mL) during surgery | 504.1±380.0 390.0 (30.0 - 1900.0) n=35 | 504.1±276.2 420.0 (30.0 - 1207.0) n=41 | 0.54 |
| Hemofiltration during ECC (mL) during surgery | 514.0±1063.4 0.0 (0.0 - 4840.0) n=35 | 117.1±469.0 0.0 (0.0 - 2600.0) n=41 | 0.017 |
| Other fluids lost (mL) during surgery | 234.7±322.1 200.0 (0.0 - 1800.0) n=35 | 173.3±190.8 200.0 (0.0 - 900.0) n=41 | 0.30 |
| Net fluid balance (mL) during surgery | 1317.8±1689.4 1275.0 (-1711.0 - 6406.0) n=35 | 2403.5±1688.9 2292.0 (-1917.0 - 6331.0) n=41 | 0.0060 |
| ASAT (µkat/L) during induction of anesthesia | 0.4±0.2 0.4 (0.2 - 1.0) n=34 | 0.5±0.2 0.4 (0.3 - 1.3) n=41 | 0.44 |
| ALAT (µkat/L) during induction of anesthesia | 0.4±0.2 0.4 (0.1 - 1.1) n=34 | 0.5±0.5 0.4 (0.1 - 3.2) n=41 | 0.21 |
| Inotropes given during 0-24h post surgery | 1 (2.9%) | 3 (7.3%) | 0.62 |
| Vasopressors given during 0-24h post surgery | 16 (45.7%) | 17 (41.5%) | 0.82 |
| Diuretics given during 0-24h post surgery | 34 (97.1%) | 37 (90.2%) | 0.37 |
| Crystalloids (mL) during 0-24h post surgery | 3717.8±1634.4 4193.0 (600.0 - 6768.0) n=35 | 4027.6±2351.4 3534.0 (0.0 - 8600.0) n=41 | 0.78 |
| Colloids (mL) during 0-24h post surgery | 204.3±278.8 100.0 (0.0 - 1100.0) n=35 | 107.3±149.0 0.0 (0.0 - 500.0) n=41 | 0.14 |
| Erythrocytes (mL) during 0-24h post surgery | 258.0±415.0 0.0 (0.0 - 1998.0) n=35 | 129.6±240.3 0.0 (0.0 - 1061.0) n=41 | 0.12 |
| Erythrocytes given post surgery | 16 (45.7%) | 12 (29.3%) | 0.16 |
| Plasma (mL) during 0-24h post surgery | 200.5±522.8 0.0 (0.0 - 2250.0) n=35 | 135.2±452.7 0.0 (0.0 - 2400.0) n=41 | 0.37 |
| Plasma given post surgery | 7 (20.0%) | 5 (12.2%) | 0.53 |
| Thrombocytes (mL) during 0-24h post surgery | 48.5±157.6 0.0 (0.0 - 750.0) n=35 | 40.9±135.0 0.0 (0.0 - 586.0) n=41 | 0.85 |
| Thrombocytes given post surgery | 4 (11.4%) | 4 (9.8%) | 1.00 |
| Fibrinogen (g) during 0-24h post surgery | 1.0±1.4 0.0 (0.0 - 5.0) n=17 | 0.1±0.5 0.0 (0.0 - 2.0) n=14 | 0.028 |
| Fibrinogen given during 0-24h post surgery | 8 (47.1%) | 1 (7.1%) | 0.021 |
| Factor II, VII, IX and X (Ocplex, Confidex) (IU) during 0-24h post surgery | 88.2±264.3 0.0 (0.0 - 1000.0) n=17 | 71.4±267.3 0.0 (0.0 - 1000.0) n=14 | 0.73 |
| Factor II, VII, IX and X (Ocplex, Confidex) given during 0-24h post surgery | 2 (11.8%) | 1 (7.1%) | 1.00 |
| Factor VII concentrate (mg) during 0-24h post surgery | 0.0±0.0 0.0 (0.0 - 0.0) n=17 | 0.0±0.0 0.0 (0.0 - 0.0) n=14 |  |
| Factor VII concentrate given during 0-24h post surgery | 0(0.0%) | 0(0.0%) |  |
| Bleeding (mL) during 0-24h post surgery | 911.1±555.2 750.0 (375.0 - 3030.0) n=35 | 840.2±654.0 560.0 (225.0 - 2900.0) n=41 | 0.07 |
| Urine production (mL) during 0-24h post surgery | 2288.6±869.6 2050.0 (895.0 - 4420.0) n=35 | 2544.9±975.3 2340.0 (1270.0 - 5050.0) n=41 | 0.27 |
| Other fluids lost (mL) during 0-24h post surgery | 174.1±308.0 0.0 (0.0 - 799.0) n=35 | 149.7±298.2 0.0 (0.0 - 916.0) n=41 | 0.71 |
| Net fluid balance (mL) during 0-24h post surgery | 1055.3±1657.0 1078.0 (-3683.0 - 4374.0) n=35 | 905.7±2109.6 987.0 (-5420.0 - 5060.0) n=41 | 0.78 |
| ASAT (µkat/L) during 0-24h post surgery | 1.5±1.3 1.2 (0.6 - 6.8) n=35 | 1.1±0.6 1.0 (0.4 - 2.9) n=41 | 0.13 |
| ALAT (µkat/L) during 0-24h post surgery | 0.5±0.2 0.4 (0.1 - 1.2) n=35 | 0.6±0.5 0.4 (0.2 - 2.8) n=41 | 0.55 |
| INR during 0-24h post surgery (24±3h post ECC) | 1.3±0.3 1.2 (1.0 - 2.8) n=33 | 1.3±0.2 1.3 (1.0 - 1.6) n=38 | 0.29 |
| APTT (s) during 0-24h post surgery (24±3h post ECC) | 28.5±5.0 27.7 (24.0 - 50.0) n=32 | 27.9±3.2 28.0 (23.0 - 41.0) n=37 | 0.91 |
| Fibrinogen (g/L) during 0-24h post surgery (24±3h post ECC) | 4.2±0.4 4.1 (3.3 - 5.1) n=32 | 3.9±0.8 4.1 (1.8 - 6.4) n=37 | 0.14 |
| Thrombocytes (10^9/L) during 0-24h post surgery (24±3h post ECC) | 175.6±73.4 148.0 (62.0 - 421.0) n=35 | 162.3±49.5 151.0 (92.0 - 311.0) n=41 | 0.59 |
| Inotropes given during 24-48h post surgery | 2 (5.7%) | 3 (7.3%) | 1.00 |
| Vasopressors given during 24-48h post surgery | 3 (8.6%) | 5 (12.2%) | 0.72 |
| Fibrinogen (g) during 24-48h post surgery | 0.0±0.0 0.0 (0.0 - 0.0) n=11 | 0.0±0.0 0.0 (0.0 - 0.0) n=14 |  |
| Fibrinogen given during 24-48h post surgery | 0(0.0%) | 0(0.0%) |  |
| Factor II, VII, IX and X (Ocplex, Confidex) (IU) during 24-48h post surgery | 0.0±0.0 0.0 (0.0 - 0.0) n=11 | 0.0±0.0 0.0 (0.0 - 0.0) n=14 |  |
| Factor II, VII, IX and X (Ocplex, Confidex) given during 24-48h post surgery | 0(0.0%) | 0(0.0%) |  |
| Factor VII concentrate (mg) during 24-48h post surgery | 0.0±0.0 0.0 (0.0 - 0.0) n=11 | 0.0±0.0 0.0 (0.0 - 0.0) n=14 |  |
| Factor VII concentrate given during 24-48h post surgery | 0(0.0%) | 0(0.0%) |  |
| Inotropes given during 48-72h post surgery | 2 (5.7%) | 2 (4.9%) | 1.00 |
| Vasopressors given during 48-72h post surgery | 2 (5.7%) | 2 (4.9%) | 1.00 |
| Fibrinogen (g) during 48-72h post surgery | 0.0±0.0 0.0 (0.0 - 0.0) n=8 | 0.0±0.0 0.0 (0.0 - 0.0) n=7 | 1.00 |
| Fibrinogen given during 48-72h post surgery | 0(0.0%) | 0(0.0%) |  |
| Factor II, VII, IX and X (Ocplex, Confidex) (IU) during 48-72h post surgery | 0.0±0.0 0.0 (0.0 - 0.0) n=8 | 0.0±0.0 0.0 (0.0 - 0.0) n=7 |  |
| Factor II, VII, IX and X (Ocplex, Confidex) given during 48-72h post surgery | 0(0.0%) | 0(0.0%) |  |
| Factor VII concentrate (mg) during 48-72h post surgery | 0.0±0.0 0.0 (0.0 - 0.0) n=8 | 0.0±0.0 0.0 (0.0 - 0.0) n=7 |  |
| Factor VII concentrate given during 48-72h post surgery | 0(0.0%) | 0(0.0%) |  |
| Inotropes given during 72-96h post surgery | 1 (2.9%) | 0 (0.0%) | 0.46 |
| Vasopressors given during 72-96h post surgery | 0 (0.0%) | 1 (2.4%) | 1.00 |
| Fibrinogen (g) during 0-24h post surgery | 1.0±1.4 0.0 (0.0 - 5.0) n=17 | 0.1±0.5 0.0 (0.0 - 2.0) n=14 | 0.028 |
| Fibrinogen given during 72-96h post surgery | 0(0.0%) | 0(0.0%) |  |
| Factor II, VII, IX and X (Ocplex, Confidex) (IU) during 72-96h post surgery | 0.0±0.0 0.0 (0.0 - 0.0) n=4 | 0.0±0.0 0.0 (0.0 - 0.0) n=3 |  |
| Factor II, VII, IX and X (Ocplex, Confidex) given during 72-96h post surgery | 0(0.0%) | 0(0.0%) |  |
| Factor VII concentrate (mg) during 72-96h post surgery | 0.0±0.0 0.0 (0.0 - 0.0) n=4 | 0.0±0.0 0.0 (0.0 - 0.0) n=3 |  |
| Factor VII concentrate given during 72-96h post surgery | 0(0.0%) | 0(0.0%) |  |
| Data are presented as mean±standard deviation, median (range) and number of observations, or number (percentage). For test between two groups Fisher's exact test was used for dichotomous variables, and Mann-Whitney U-test for continuous variables. | | | |

# Table 8.1 Overall summary of adverse events by actual treatment group (Safety population)

|  | **PrimeECC N=42** | **SOC N=50** | **p-value** |
| --- | --- | --- | --- |
| Any AE | 23 (54.8%) | 23 (46.0%) | 0.53 |
| Any SAE | 10 (23.8%) | 14 (28.0%) | 0.81 |
| Any AE related to procedure | 14 (33.3%) | 14 (28.0%) | 0.65 |
| Any AE related to device | 13 (31.0%) | 12 (24.0%) | 0.49 |
| Any AE leading to death | 2 (4.8%) | 1 (2.0%) | 0.59 |
| Data are presented as number (percentage). | | | |

# Table 8.2 Incidence of adverse events by SOC and PT by actual treatment group (Safety population)

|  | **PrimeECC N=42** | | **SOC N=50** | |
| --- | --- | --- | --- | --- |
|  | **n Events** | **n (%) Patients with events** | **n Events** | **n (%) Patients with events** |
| **Blood and lymphatic system disorders** | **3** | **3 (7.1%)** | **6** | **5 (10.0%)** |
| Blood loss anaemia | 0 | 0 (0.0%) | 1 | 1 (2.0%) |
| Coagulopathy | 1 | 1 (2.4%) | 0 | 0 (0.0%) |
| Factor I deficiency | 1 | 1 (2.4%) | 0 | 0 (0.0%) |
| Hypofibrinogenamina | 1 | 1 (2.4%) | 5 | 5 (10.0%) |
| **Cardiac disorders** | **8** | **7 (16.7%)** | **11** | **10 (20.0%)** |
| Atrial fibrillation | 4 | 4 (9.5%) | 5 | 5 (10.0%) |
| Cardiac failure | 1 | 1 (2.4%) | 1 | 1 (2.0%) |
| Cardiac failure acute | 1 | 1 (2.4%) | 0 | 0 (0.0%) |
| Cardiac tamponade | 0 | 0 (0.0%) | 2 | 2 (4.0%) |
| Mitral valve incompetence | 0 | 0 (0.0%) | 1 | 1 (2.0%) |
| Right ventricular failure | 1 | 1 (2.4%) | 1 | 1 (2.0%) |
| Ventricular arrhythmia | 1 | 1 (2.4%) | 1 | 1 (2.0%) |
| **General disorders and administration site conditions** | **1** | **1 (2.4%)** | **0** | **0 (0.0%)** |
| Paravalvular regurgitation | 1 | 1 (2.4%) | 0 | 0 (0.0%) |
| **General disorders and administration site conditions** | **2** | **2 (4.8%)** | **3** | **3 (6.0%)** |
| Oedema peripheral | 1 | 1 (2.4%) | 2 | 2 (4.0%) |
| Prosthetic cardiac valve regurgitation | 1 | 1 (2.4%) | 0 | 0 (0.0%) |
| Pyrexia | 0 | 0 (0.0%) | 1 | 1 (2.0%) |
| **Infections and infestations** | **4** | **4 (9.5%)** | **0** | **0 (0.0%)** |
| Endocarditis | 1 | 1 (2.4%) | 0 | 0 (0.0%) |
| Fungal skin infection | 1 | 1 (2.4%) | 0 | 0 (0.0%) |
| Pneumonia | 1 | 1 (2.4%) | 0 | 0 (0.0%) |
| Pneumonia bacterial | 1 | 1 (2.4%) | 0 | 0 (0.0%) |
| **Injury, poisoning and procedural complications** | **8** | **8 (19.0%)** | **15** | **13 (26.0%)** |
| Anemia postoperative | 0 | 0 (0.0%) | 2 | 2 (4.0%) |
| Aortic injury | 0 | 0 (0.0%) | 1 | 1 (2.0%) |
| Factor I deficiency | 0 | 0 (0.0%) | 1 | 1 (2.0%) |
| Mechanical ventilation complication | 0 | 0 (0.0%) | 1 | 1 (2.0%) |
| Post procedural haemorrhage | 3 | 3 (7.1%) | 4 | 4 (8.0%) |
| Post procedural hypotension | 1 | 1 (2.4%) | 0 | 0 (0.0%) |
| Post procedural stroke | 0 | 0 (0.0%) | 1 | 1 (2.0%) |
| Post-procedural hypotension | 0 | 0 (0.0%) | 1 | 1 (2.0%) |
| Postoperative respiratory failure | 3 | 3 (7.1%) | 1 | 1 (2.0%) |
| Procedural vomiting | 0 | 0 (0.0%) | 1 | 1 (2.0%) |
| Vasoplegia syndrome | 1 | 1 (2.4%) | 1 | 1 (2.0%) |
| Wound secretion | 0 | 0 (0.0%) | 1 | 1 (2.0%) |
| **Investigations** | **1** | **1 (2.4%)** | **0** | **0 (0.0%)** |
| Myocardial necrosis marker increased | 1 | 1 (2.4%) | 0 | 0 (0.0%) |
| **Nervous system disorders** | **1** | **1 (2.4%)** | **0** | **0 (0.0%)** |
| Seizure | 1 | 1 (2.4%) | 0 | 0 (0.0%) |
| **Psychiatric disorders** | **1** | **1 (2.4%)** | **3** | **3 (6.0%)** |
| Delirium | 1 | 1 (2.4%) | 3 | 3 (6.0%) |
| **Renal and urinary disorders** | **7** | **6 (14.3%)** | **8** | **7 (14.0%)** |
| Acute kidney injury | 7 | 7 (16.7%) | 8 | 8 (16.0%) |
| **Respiratory, thoracic and mediastinal disorders** | **3** | **3 (7.1%)** | **1** | **1 (2.0%)** |
| Pleural effusion | 0 | 0 (0.0%) | 1 | 1 (2.0%) |
| Pneumothorax | 2 | 2 (4.8%) | 0 | 0 (0.0%) |
| Respiratory failure | 1 | 1 (2.4%) | 0 | 0 (0.0%) |
| **Vascular disorders** | **0** | **0 (0.0%)** | **1** | **1 (2.0%)** |
| Aortic dissection | 0 | 0 (0.0%) | 1 | 1 (2.0%) |
| Data are presented as number (percentage). | | | | |

# Table 8.3 Incidence of serious adverse events by SOC and PT by actual treatment group (Safety population)

|  | **PrimeECC N=42** | | **SOC N=50** | |
| --- | --- | --- | --- | --- |
|  | **n Events** | **n (%) Patients with events** | **n Events** | **n (%) Patients with events** |
| **Blood and lymphatic system disorders** | **1** | **1 (2.4%)** | **5** | **5 (10.0%)** |
| Factor I deficiency | 1 | 1 (2.4%) | 0 | 0 (0.0%) |
| Hypofibrinogenamina | 0 | 0 (0.0%) | 5 | 5 (10.0%) |
| **Cardiac disorders** | **2** | **2 (4.8%)** | **2** | **2 (4.0%)** |
| Cardiac failure | 1 | 1 (2.4%) | 0 | 0 (0.0%) |
| Cardiac failure acute | 1 | 1 (2.4%) | 0 | 0 (0.0%) |
| Cardiac tamponade | 0 | 0 (0.0%) | 2 | 2 (4.0%) |
| **General disorders and administration site conditions** | **1** | **1 (2.4%)** | **0** | **0 (0.0%)** |
| Paravalvular regurgitation | 1 | 1 (2.4%) | 0 | 0 (0.0%) |
| **Injury, poisoning and procedural complications** | **3** | **3 (7.1%)** | **9** | **8 (16.0%)** |
| Anemia postoperative | 0 | 0 (0.0%) | 2 | 2 (4.0%) |
| Factor I deficiency | 0 | 0 (0.0%) | 1 | 1 (2.0%) |
| Post procedural haemorrhage | 1 | 1 (2.4%) | 3 | 3 (6.0%) |
| Post procedural stroke | 0 | 0 (0.0%) | 1 | 1 (2.0%) |
| Post-procedural hypotension | 0 | 0 (0.0%) | 1 | 1 (2.0%) |
| Postoperative respiratory failure | 2 | 2 (4.8%) | 1 | 1 (2.0%) |
| **Nervous system disorders** | **1** | **1 (2.4%)** | **0** | **0 (0.0%)** |
| Seizure | 1 | 1 (2.4%) | 0 | 0 (0.0%) |
| **Renal and urinary disorders** | **5** | **5 (11.9%)** | **2** | **2 (4.0%)** |
| Acute kidney injury | 5 | 5 (11.9%) | 2 | 2 (4.0%) |
| **Vascular disorders** | **0** | **0 (0.0%)** | **1** | **1 (2.0%)** |
| Aortic dissection | 0 | 0 (0.0%) | 1 | 1 (2.0%) |
| Data are presented as number (percentage). | | | | |

# Table 8.4 Incidence of adverse events leading to death by SOC and PT by actual treatment group (Safety population)

|  | **PrimeECC N=42** | | **SOC N=50** | |
| --- | --- | --- | --- | --- |
|  | **n Events** | **n (%) Patients with events** | **n Events** | **n (%) Patients with events** |
| **Cardiac disorders** | **1** | **1 (2.4%)** | **0** | **0 (0.0%)** |
| Cardiac failure acute | 1 | 1 (2.4%) | 0 | 0 (0.0%) |
| **General disorders and administration site conditions** | **1** | **1 (2.4%)** | **0** | **0 (0.0%)** |
| Paravalvular regurgitation | 1 | 1 (2.4%) | 0 | 0 (0.0%) |
| **Vascular disorders** | **0** | **0 (0.0%)** | **1** | **1 (2.0%)** |
| Aortic dissection | 0 | 0 (0.0%) | 1 | 1 (2.0%) |
| Data are presented as number (percentage). | | | | |
