# Supplementary material for: Dextran vs. Crystalloid Priming Solution in Cardiac Surgery: A Randomized Trial on Acute Kidney Injury
Source: Acta Anaesthesiol Scand. 2025 Oct 27;70(1):e70139. doi: 10.1111/aas.70139 (PMC12558647; doi:10.1111/aas.70139)
Supplement: Supplementary file 3 — Data S3: Supporting Information. [file AAS-70-0-s002.docx]

| Supplemental Table 1. Intraoperative data |  |  | |  |
| --- | --- | --- | --- | --- |
|  | **Dextran (n=43)** | | **SOC (n=49)** | **p** |
| Cardiac Pocedure |  |  | | 0.06 |
| CABG | 18 (41.9) | 14 (28.6) | |  |
| Valvular surgery | 7 (16.3) | 17 (34.7) | |  |
| CABG + Valvular surgery | 16 (37.2) | 11 (22.4) | |  |
| Aorta Surgery | 1 (2.3) | 1 (2.0) | |  |
| Other | 1 (2.3) | 1 (2.0) | |  |
| Operative priority |  |  | | 0.09 |
| Elective | 23 (53.5) | 35 (71.4) | |  |
| Urgent | 20 (46.5) | 14 (28.6) | |  |
| CPB time, hours | 1.8 [0.8 – 7.3] | 1.4 [0.9 – 8.4] | | 0.09 |
| Clamp time, hours | 1.3 [0.6 – 4.5] | 1.1 [0.5 – 4.2] | | 0.36 |
| Data are presented as median with range or number of observations (percentage). Abbreviations: CABG, Coronary Artery Bypass Graft; CPB, Cardiopulmonary Bypass; SOC, Standard of Care. Statistical Comparisons are Fisher’s Exact Test for categorical variables and Mann-Whitney U-test for continuous variables | | | | |
|  | |  | |  |

| Supplemental Table 2. Postoperative fluids and medication, first 24 hours | | |  |
| --- | --- | --- | --- |
|  | **Dextran (n=43)** | **SOC (n=49)** | **p** |
| Vasopressor use | 19 (45) | 20 (42) | 0.83 |
| Net fluid balance, ml | 1115[-3683 – 4374] | 801[-5420 – 5060] | 0.35 |
| Crystalloids, ml | 4276[600 – 6768] | 3549[0 – 8600] | 0.92 |
| Colloids, ml | 100[0 – 1100] | 0[0 – 1000] | 0.24 |
| Urine output, ml | 2250[895 – 4420] | 2365[1270 – 5050] | 0.25 |
| Bleeding, ml | 750[275 – 3030] | 565[225 – 2900] | 0.14 |
| Erythrocytes, ml | 0[0 – 1998] | 0[0 – 1061] | 0.15 |
| Plasma, ml | 0[0 – 2250] | 0[0 – 2400] | 0.37 |
| Thromocytes, ml | 0[0 – 750] | 0[0 – 586] | 0.84 |
| Data are presented as median with range or number of observations (percentage). Abbreviations: SOC Standard of Care. Statistical Comparisons are Fisher’s Exact Test for categorical variables and Mann-Whitney U-test for continuous variables. | | | |

**The following parameters were included in AKI-risk score model:**

Age (<60, 60-74, ≥75)

Sex (male/female)

BMI (kg/m2) (<20, 20–24.9, 25–29.9, 30–34.9, ≥35)

Smoking habits (never, ex-smoker, current)

Diabetes mellitus (yes/no)

Peripheral vascular disease (yes/no)

Hypertension (yes/no)

NYHA-class (1, 2, 3, 4)

Preoperative haemoglobin ((g/dL): (<10, 10–11.9, ≥12))

Preoperative eGFR (Cockroft–Gault formula, ml/min): ≥90, 60–89.9, 30–59.9, <30

Time from catheterism to surgery (within 24h, >24h this admission, >24 previous admission)

Triple vessel disease (yes/no)

LV ejection fraction (≥50%, 30–49%, <30%)

Operative priority (elective, urgent, emergency)

Type of surgery (CABG, single valve, CABG + valve, other/multiple)

**The PP population excluded patients with major protocol violations. These violations were:**

*AKI risk below 30: dextran n=2, control n=3*

*One postoperative creatinine value missing: dextran n=3, control n=5*

*Death during surgery: dextran n=2, control n=0*

*Incorrect treatment given (i.e. receiving wrong priming solution): dextran n=1, control =0*

*Data availability statement:*

*Upon request, we will provide de-identified individual participant data to fellow researchers. The trial data have been acquired, managed, and archived in compliance with Good Clinical Practice (GCP) and ISO14155:20 standards. Formal data sharing requests are carefully considered, taking into account the guidelines of the study centers and sponsor. Those interested in obtaining data should contact the corresponding author, providing details about the proposed research and the extent of data requirements.*

*To access the data, recipients must enter into a formal data sharing agreement outlining the conditions for release and specifying requirements for data transfer, storage, archiving and publication. Data sharing is approved if the proposed projects demonstrate a sound scientific or patient benefit rationale.*

*To uphold patient confidentiality and consent, restrictions are imposed by aggregating and anonymizing identifiable patient data. Furthermore, all indirect identifiers that could potentially lead to deductive disclosures will be systematically removed.*
